# High-Resolution ^1^H NMR Investigation of the Speciation Status of Nickel(II) and Copper(II) Ions in a Cell Culture Medium: Relevance to Their Toxicological Actions

**DOI:** 10.3390/molecules31010085

**Published:** 2025-12-24

**Authors:** Deepinder K. Kalra, Kayleigh Hunwin, Katie Hewitt, Olivia Steel, Martin Grootveld

**Affiliations:** Leicester School of Pharmacy, De Montfort University, The Gateway, Leicester LE1 9BH, UK; p2824690@alumni365.dmu.ac.uk (D.K.K.); p2421485@my365.dmu.ac.uk (K.H.); p2585316@my365.dmu.ac.uk (K.H.); p2589319@my365.dmu.ac.uk (O.S.)

**Keywords:** trace metal ions: nickel(II) and copper(II) ions, speciation, ^1^H NMR analysis, RPMI 1640 culture medium, trace metal ion toxicity

## Abstract

Copper and nickel ions play pivotal, albeit distinct, roles as essential trace elements in living systems, and primarily serve as co-factors for a range of enzymes. However, as with all trace metal ions, excessive concentrations can exert adverse toxicological properties. Interestingly, the incorporation of these in cell culture media can establish novel chemical interactions, with their speciation status markedly influencing characteristics, including cell maturation, and cellular uptake mechanisms. Thus, the primary objective of this study was to investigate and determine the speciation status (i.e., complexation) of nickel(II) and copper(II) ions by biomolecules present in RPMI 1640 mammalian cell culture medium using virtually non-invasive high-resolution proton NMR analysis, an investigation of much relevance to now routine studies of their toxicological actions towards cultured cells. Samples of the above aqueous culture medium were ^1^H NMR-titrated with increasing added concentrations of 71–670 µmol/L Ni(II)(aq.), and 0.71–6.7, 7.1–67 and 71–670 µmol/L Cu(II)(aq.), in duplicate or triplicate. ^1^H NMR spectra were acquired on a JEOL ECZ-600 spectrometer at 298 K. Results demonstrated that addition of increasing concentrations of Ni(II) and Cu(II) ions to the culture medium led to the selective broadening of a series of biomolecule resonances, results demonstrating their complexation by these agents. The most important complexants for Ni(II) were histidine > glutamine > acetate ≈ methionine ≈ lysine ≈ threonine ≈ branched-chain amino acids (BCAAs) > asparagine ≈ aspartate > tyrosine ≈ tryptophan, whereas for Cu(II) they were found to be histidine > glutamine > phenylalanine ≈ tyrosine ≈ nearly all remaining aliphatic metabolites (particularly the wealth of amino acids detectable) > 4-hydroxyphenylacetate (trace culture medium contaminant), in these orders. However, Cu(II) had the ability to influence the linewidths of these signals at much lower added levels (≤7 µmol/L) than that of Ni(II), the broadening effects of the latter occurring at concentrations which were approximately 10-fold greater. Virtually all of these added metal ion-induced resonance modifications were, as expected, reversible on addition of equivalent or excess levels of the chelator EDTA. From this study, changes in the co-ordination sphere of metal ions in physiological environments can give rise to marked modifications in their physicochemical properties (e.g., redox potentials, electronic charges, the potential catalytic generation of reactive oxygen species (ROS), and cell membrane passages). Moreover, given that the above metabolites may also function as potent hydroxyl radical (^●^OH) scavengers, these findings suggest that generation of this aggressively reactive oxidant directly from Cu(II) and Ni(II) ions in physiologically-relevant complexes may be scavenged in a ‘site-dependent’ manner. This study is of further relevance to trace metal ion research in general since it enhances our understanding of the nature of their interactions with culture medium biomolecules, and therefore provides valuable information regarding their overall chemical and biological activities, and toxicities.

## 1. Introduction

The very broad scope of biochemical reaction processes involving numerous biomolecules comprising proteins, nucleic acids, lipids, carbohydrates and low-molecular-mass metabolites, often also require essential metal ions. Indeed, when associated with biomolecules, transition and other metal ions usually play structural control (e.g., steadying the macromolecular structures of proteins), and/or catalytic roles (i.e., representing a critical part of the active sites of enzymes), as well as influencing proteins or cellular membranes in order to regulate cell metabolism. However, administration or ingestion of elevated doses of these metal ions can engender toxicological actions in biological systems. For example, trace metal ions, including those of copper and nickel, transpire naturally in the environment and are important for the continued existence and longevity of humans and animals by being part of a healthy diet, or alternatively as nutritional additives. Normal function requires that such metal ion levels be upheld within a suitable range; indeed, lower environmentally available concentrations might lead to a nutritional deficiencies, whilst higher levels might present some risk of adverse health effects. For example, copper ions contribute to redox reactions in energy metabolism. Having copper deficiency alters important metabolic processes, and higher exposures might result in neurotoxicity, a process that may give rise to life-changing neurological diseases such as Alzheimer’s and Parkinson’s diseases, as well as cancer [1]. Furthermore, the presence of these metal ions in a cell culture medium plays a crucial role in influencing cell maturation, metabolism, and the quality of cellular products; indeed, they may readily react with biomolecular complexants present in the medium to form corresponding metal ion complexes, and their species generated in this manner (commonly known as ‘speciation’) has the potential to greatly affect their chemical reactivity characteristics, and this influences cellular uptake mechanisms and other physiological functions [2]. Hence, a watchful ‘steady-state’ concentration level of these trace metal ions is imperative for upholding the body’s homeostasis. Notably, the overall concentration level and speciation status are equally crucial for determining nutritional adequacy, and also the development of forecasts of bioavailability for a dependable risk assessment approach.

Therefore, the primary objective of this study was to investigate and determine the speciation status (i.e., complexation) of copper(II) and nickel(II) ions by biomolecules present in RPMI 1640 culture medium using high-resolution, high-field proton nuclear magnetic resonance (^1^H NMR) spectroscopy. Such investigations are also of much relevance to experiments focused on the potential toxicity of these and other metal ions towards a range of cell types which are routinely cultured in media supplemented with increasing levels of such toxins. Clearly, any toxic effects found in these experiments are often incorrectly assigned to the ‘added’ form of the metal ion, and not on the species which is generated in the culture medium through usually very thermodynamically favourable complexation reactions.

### 1.1. Biological Roles of Trace Metal Ions—Copper and Nickel Ions

Copper(I) and (II) ions are important trace metal ions, and essential to all human beings as a food mineral (intake of 1 to 100 mg per day is required by adults) [3]. It acts as a catalytic co-factor in a range of biological processes, including free radical neutralisation (Cu/Zn superoxide dismutase (SOD)-1), neurological development, electron transport pathways, (e.g., cytochrome-c oxidase complexes), haemoglobin production, iron homeostasis (ceruloplasmin) and glucose metabolism, as well as forming cross-linkages in collagen, and keratin fibres in hair. Nevertheless, the disequilibrium of copper ions via reduced absorption or excretion culminates in deficiency or toxicity. For example, Menkes disease is a hereditary neurodegenerative illness connected with copper metabolism that results from a significant change in intracellular copper transport in humans [4].

Nickel(II) also has a wide range of physiological functions, including serving as a blood production stimulant, and encouraging the restoration of red blood cells. It is present in enzymes such as urease (a dinuclear Ni(II) ion-containing metalloenzyme), and catalyses the terminal phase in methane generation (via methanogenic bacteria). It also has a wide range of industrial applications in numerous manufacturing and refining operations [5]. Nevertheless, this transition metal ion can give rise to quite severe inflammation and skin allergies, and the inhalation of aerosols of Ni(II) compounds (>0.5 g) for longer periods has been associated with lung cancer, pulmonary fibrosis, and kidney and cardiovascular diseases in exposed nickel industry workers, as well as amongst the population in general [6].

^1^H NMR spectroscopy is a highly robust tool for revealing and determining the nature and amounts of magnetically active nuclei, including those of hydrogen (which are commonly referred to as protons present in analytes), benefits which provide valuable information regarding the structure of any molecule containing such ^1^H nuclei. Hence, protons present in a molecule act differently according to their adjacent chemical environment(s), permitting researchers to determine detailed molecular structures in a very wide range of analytes [7]. In comparison to the analytical methods such as electron microscopy, mass spectrometry (usually in tandem with high-performance liquid or gas chromatography), along with a myriad of alternative bioanalytical laboratory techniques, ^1^H NMR analysis provides numerous benefits for examining the complexation (speciation) status of metal ions in biological samples by analysing specific resonance broadenings and/or chemical shift changes associated with ligand-exchange processes and/or paramagnetism when arising from experiments featuring their addition to the medium investigated, e.g., biofluid, culture medium, etc. [8].

The unpaired electrons of transition metal ions interact with nuclear spins, and substantially complicate the ^1^H NMR profiles of biofluids and related media, and significantly or markedly alter relaxation times, a process giving rise to broadened resonances therein. However, as demonstrated previously, and also here, careful control of the levels of paramagnetic metal ions added to such matrices allows evaluations of their complexation status therein [8,9,10,11,12,13]. A further challenge, however, is that the unpaired electrons of transition metal ions can result in large or very large modifications in the chemical shift values of biomolecular complexants, which renders their detection and two-dimensional (2D) correlation somewhat arduous.

### 1.2. Speciation of Metal Ions in Biosamples

‘Speciation’ is concerned with the distribution of an element or metal ion amongst distinct chemical species in a given system [14]. Each transition metal ion generates diverse species based on factors including added metal ion concentration level, its oxidation state (reflected by redox potential of the couple involved), temperature, pH, ionic strength, ligand availability, and other competing complexing molecules present in its immediate environment. This information is vital, since the precise chemical species (i.e., the nature of the compound—organic or inorganic form) of an element in which it is present can substantially influence its toxicity, reactivity, bioavailability, and mobility [15]. In addition, variations in affinities of transition metal ions in different oxidation states, for example, with ligands containing ‘hard’ and/or ‘soft’ donor atoms, are crucial in enabling cellular uptake and transfer reactions. For example, the less harmful forms of mercury are the inorganic ones, while the most lethal ones are its alkylated species because of their lipid solubility traits, and also their ability to easily penetrate cell membranes. An additional illustration is chromium, which in its +(III) oxidation state plays a significant role in glucose metabolism in humans, but its (VI) oxidation state is exceedingly harmful [16], and acts as a powerful oxidant. If a metal ion–ligand complex has zero charge, it may precipitate as a solid matrix, for example, Fe(OH)_3_ (ferrihydrite, an inorganic hydroxide precipitate), Ni(OH)_2_ (green precipitate) and Cu(OH)_2_ (blue precipitate), which at high pH values (>7) are examples of solids that could form in culture growth media) [2].

Cu(II) ions are known to tether robustly with a wide range of biomolecules, including those with carboxylato, amino and thiolo functions, which have the greatest affinities for this metal ion. Moreover, as we might expect, the complexation of Cu(II) ions with bioavailable ligands and/or chelators is strongly pH-dependent, since the protonation of these ligands decreases their capacity to complex it [17]. The introduction of metal ions at more elevated concentration levels than those required for the generation of a 1:1 complex species can change the composition and stability of the medium drastically, in view of their interactions with additional metabolites therein.

Such Cu(II) complexes can activate oxidative stress, disturb enzyme function, and impede nutrient availabilities. Selected redox-active metal ions, including those of copper and nickel, can form coordination compounds with different ligands, processes disrupting normal cellular functions. Indeed, in the case of copper ions, this may lead to the generation of reactive oxygen species (ROS), most especially the highly toxic and aggressively reactive hydroxyl radical (^●^OH) via Fenton reactions; this process impedes the viabilities and activities of cells by causing oxidative damage. This eventually results in damage to DNA and its associated repair mechanisms upon binding to histones, which play a key role in the breaking of DNA strands. In principle, Cu(II) can oxidise electron-donating biomolecules such as L-cysteine, creating a disulphide linkage and liberating Cu(I) ion. Following contact with H_2_O_2_, the reduced form of these ions liberate ^●^OH radicals (Equation 1), restoring the oxidised form (Cu(II) ion for the Cu(II):Cu(I) redox couple), which again may participate in Fenton and Fenton-type reactions [18]. Although Ni(II) is also redox-active, albeit to a much lesser extent, it can, at least in principle, also affect cellular redox balances.

M^n+^ + H_2_O_2_ → M^(n + 1)+^ + ^●^OH + OH^−^ (1)

Several findings suggest that Cu(I)- and Cu(II)-hydrogen peroxide (H_2_O_2_) admixtures can cause DNA damage via the generation of ROS [19,20,21]. This supports the theory regarding the important function of ROS in carcinogenic processes induced by metal particulates and metal ions [22].

Previously, the exact molecular characteristics of Co(II) [10], Cr(III) [11], V(III, IV and V) [12], and Al(III) [8] within osteoarthritic synovial fluid, as well as Co(II) [23] and Ni(II) [13] in human saliva, have been elucidated through the application of ^1^H NMR analysis. Similarly, the molecular nature of Cu(II) ions in inflammatory knee-joint synovial fluid has been explored using this technique [24]. Nevertheless, to date there has been little or no endeavour to reveal the molecular characteristics of the essential trace metal ions Ni(II) and Cu(II) in cell culture media, via ^1^H NMR analysis. Therefore, this study aimed to identify any coordination compounds produced from reactions between these metal ions and the various biomolecules present in a typical, frequently used culture medium (RPMI 1640). These biomolecules include amino acids, vitamins, carbohydrates (predominantly glucose), choline and organic acid anions, which in principle may impact upon the toxicity, bioavailability and general influence of these transition metal ions on cell culture processes. This study is of further relevance to trace metal ion research since it will serve to improve our understanding of how such metal ions simultaneously interact with a wide range of metabolites present in a culture medium, providing information regarding their biological activities and toxicities.

### 1.3. Objectives of the Study

Primarily, to speciate nickel(II) and copper(II) ions, i.e., investigate and determine their complexation by biomolecules, present in RPMI 1640 culture medium using ^1^H NMR spectroscopy.

Secondly, to enhance our understanding of which biomolecules have the highest affinity for these added trace metal ions; is the effectiveness of these reactions dependent on their culture medium concentrations and/or the thermodynamic stability of the complexes formed?

Finally, how does the molecular nature of metal ion-biomolecule complexes formed relate to their biological activity? what relevance is complexation status to the toxicological properties of these metal ions?

## 2. Results

**Figure 1:**
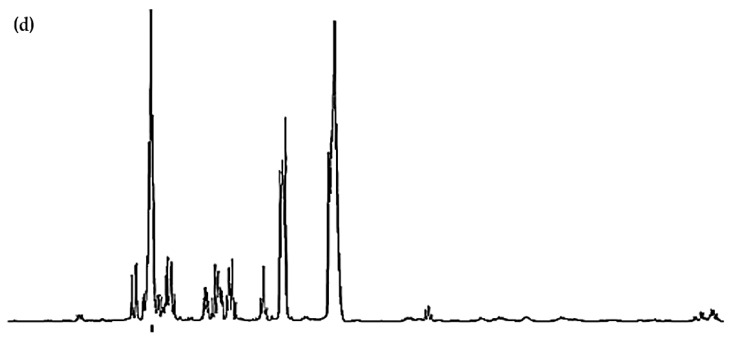

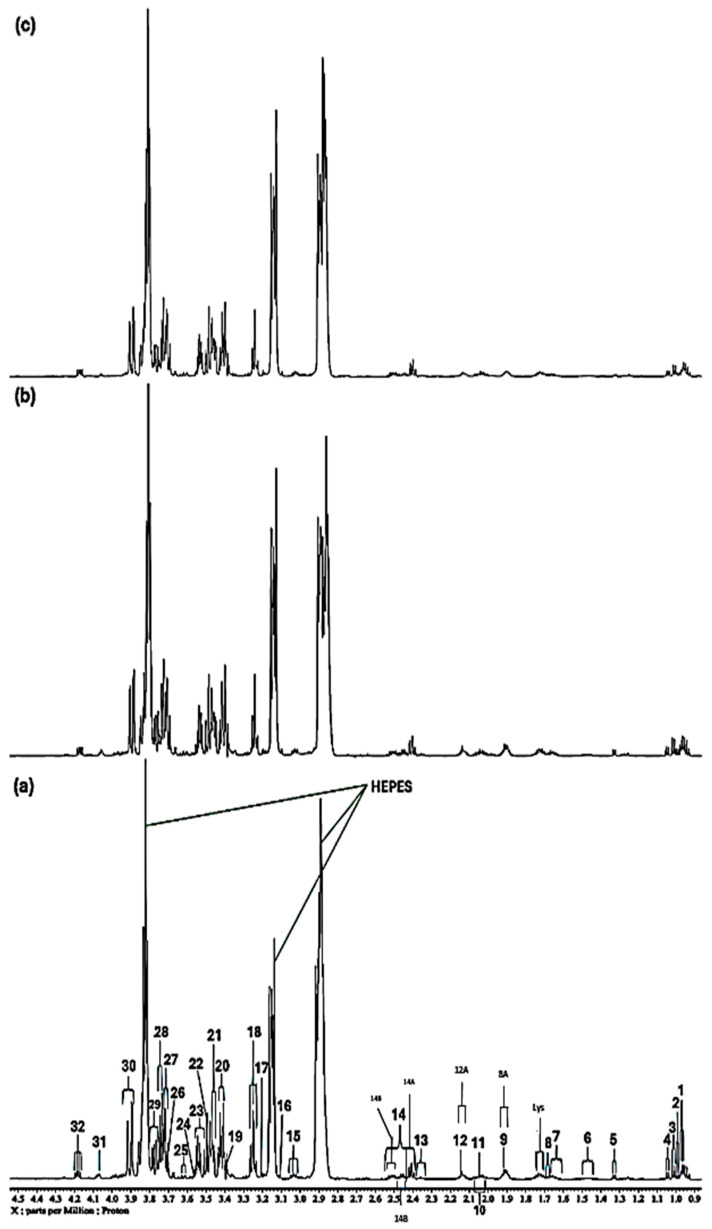
Expanded 0.86–4.54 ppm regions of the 600.17 MHz WASTED II ^1^H NMR spectra of (**a**) a control RPMI 1640 culture medium sample (untreated sample) devoid of any added Ni(II) ions, and the same sample following equilibration with Ni(II) at added final concentrations of (**b**) 71, (**c**) 140 and (**d**) 540 µmol/L. Typical spectra are shown. Assignment abbreviations: 1, Leucine-CH_3_ (*d*); 2, Leucine-CH_3_ (*d*)/Isoleucine-γ-CH_3_ (*t*) 3. Valine-CH_3_ (*d*)/Isoleucine-β-CH_3_ (*d*); 4, Valine-CH_3_ (*d*); 5, Threonine-CH_3_ (*d*); 6, Isoleucine-γ-CH_2_/Lysine-γ-CH_2_ (*m*); 7, Arginine-γ-CH_2_; 8, Leucine-CH_2_ (*m*); 8A, Lysine-β-CH_2_ (*td*); Lys, Lysine-δ-CH_2_ (*tt*); 9, Arginine-β-CH_2_ (*m*); 10, Proline-β-CH_2_ (*m*)/Proline-γ-CH_2_ (*m*)/Glutamate-β-CH_2_ (*m*)/Pyroglutamate-1/2-β-CH_2_ (m); 11, N-acetylmetabolites–NHCOCH_3_ (*s*); 11A, O-Acetylmetabolite(s)-OCOCH_3_; 12, Methionine-S-CH_3_ (sharp (*s*)); 12A, Glutamine-β-CH_2_; 13, Glutamate-γ-CH_2_ (*m*); 14, Intricate 2.36–2.54 ppm region, including 14A, Pyroglutamate-γ-CH_2_ (m), possibly superimposed on minor Pyruvate-CH_3_ (*s*) and/or Succinate-CH_2_ (*s*) resonances; 14B, Glutamine-γ-CH_2_ (*m*); 14C, Pyroglutamate-1/2-β-CH_2_; 15, Lysine-ε-CH_2_ (*t*); 16, * Dimethylsulphone SO_2_(CH_3_)_2_ (*s*); 17, Arginine-γ-CH_2_ (*m*)/Choline-N(CH_3_)_3_^+^ (*s*); 18, β-Glucose-C2H (*dd*); 19, Methanol-CH_3_; 20, α-Glucose-C4H (*dd*); 21. β-Glucose-C5H (*dt*); 22, β-Glucose-C3H (*dd*); 23, α-Glucose-C2H (*dd*); 24, Glycine-CH_2_ (*s*); 25, Valine-α-CH (*d*); 26, Unknown/Leucine-α-CH/Lysine-α-CH(*s/m/t*); 27, α-Glucose-C3H′ (*dd*); 28. β-Glucose-C6H_b_ (*dd*); 29, α-Glucose-C6H_b_ (*td*); 30. β-Glucose-C6H_a_ (*dd*); 31, Cystine-α-CH (*m*)/myo-inositol-C2H (*t*); 32, 5-Oxo-pyrrolidine-2-carboxylate (pyroglutamate)-CH_2_s (*2 x t*), a glutamate decomposition product. * Indicates tentative assignments; indicator lines represent signals arising from the HEPES buffer system (HEPES represents 4-(2-hydroxyethyl)-1-piperazine ethanesulphonate). The -CH5 and -CH6_a_ resonances of α-Glucose (δ = 3.817 and 3.823 ppm, respectively) were both obscured by the relatively intense HEPES buffer 3.83 ppm signal. Abbreviations: *s*, *d*, *dd*, *dt*, *t*, *q* and *m* denote singlet, doublet, doublet of doublets, doublet of triplets, triplet, quartet and multiplet resonance multiplicities, respectively.

**Figure 2:**
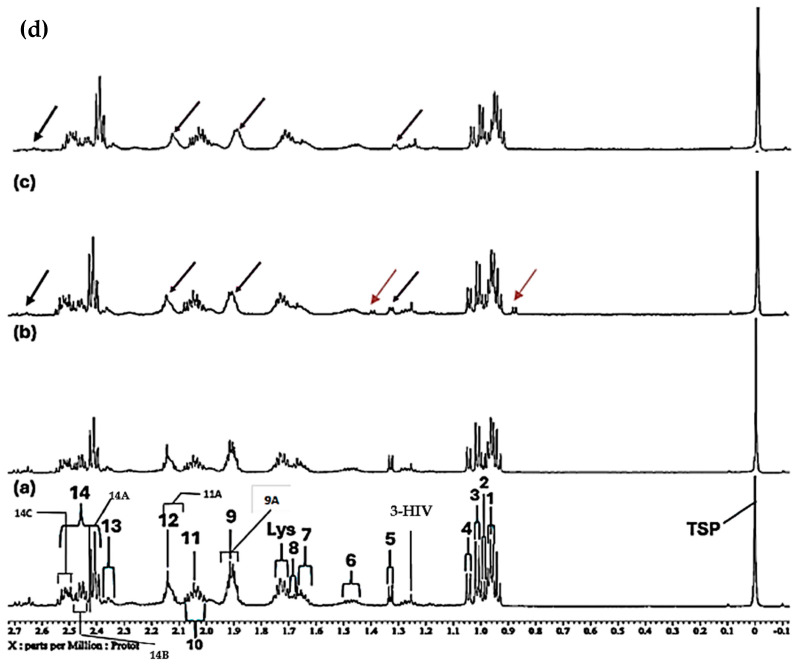
Expanded −0.10–2.72 ppm regions of the 600.17 MHz WASTED II ^1^H NMR spectra of (**a**) a control RPMI 1640 culture medium sample (untreated sample) devoid of any added Ni(II) ions, and the same sample following equilibration with Ni(II) at added final concentrations of (**b**) 71, (**c**) 140 and (**d**) 540 µmol/L. Typical spectra are shown. Assignment labels: As Figure 1, with 9A representing Arginine-γ-CH_2_, and 3-HIV a singlet resonance tentatively assigned to 3-Hydroxyisovalerate-CH_3_ protons. The very minor 2.60 (*m*) and 2.68 ppm (*dd*) signals are attributable to Methionine-γ-CH_2_ and Aspartate ½ β-CH_2_ protons. The arrows in (**c**,**d**) indicate selected resonance broadenings (purple) or the appearance of new, albeit very minor, signals resulting from the addition of Ni(II).

**Figure 3:**
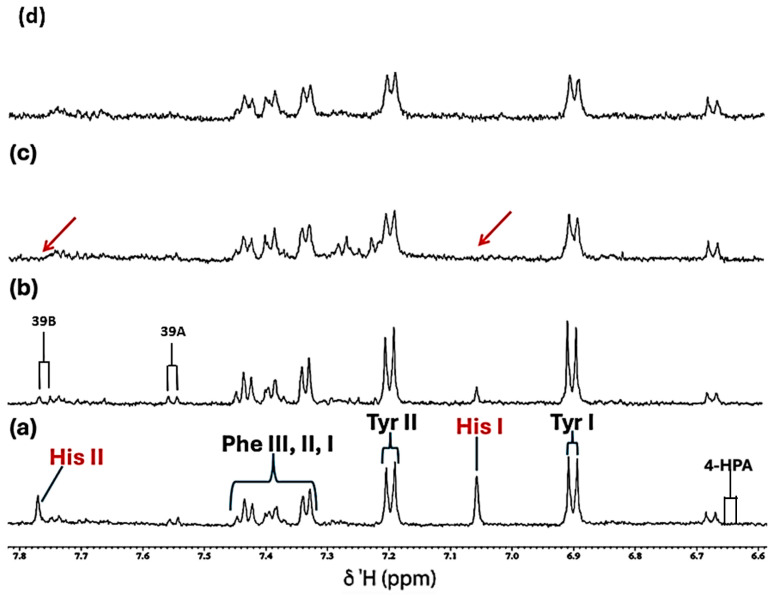
Expanded 6.58–7.84 ppm regions of the 600.17 MHz WASTED II ^1^H NMR spectra of (**a**) a control RPMI 1640 culture medium sample (untreated sample) devoid of any Ni(II) ions, and the same sample following equilibration with Ni(II) at added final concentrations of (**b**) 71, (**c**) 140 and (**d**) 540 µmol/L. Typical spectra are shown. Assignment abbreviations: 4-HPA, 4-Hydroxyphenylacetate-CH_2_ (*d*) (tentative assignment); Tyr I, Tyrosine aromatic ring-C2H/C6H (*d*); His I, Histidine imidazole ring-C5H (*s*); Tyr II, Tyrosine aromatic ring-C3H/C5H (*d*); Phe 1, Phenylalanine aromatic ring-C2H/C6H (*m*); Phe II, Phenylalanine aromatic ring-C4H (*m*); Phe III, Phenylalanine aromatic ring-C3H/C5H (*m*); 39A, Tryptophan aromatic ring-C2H (*d*); 39B, Tryptophan-aromatic ring-C3H (*d*); His II, Histidine imidazole ring-C3H (*s*). The red arrows in (**c**) indicate the broadening-mediated disappearance of the two imidazole ring histidine resonances with added Ni(II).

Evidence for the molecular nature of low-molecular-mass Ni(II) and Cu(II) complexes within the RPMI 1640 culture medium was provided by high-field ^1^H NMR spectroscopy. As previously documented by Jinks et al., 2024 [25], ^1^H NMR spectral analysis of this mammalian cell culture medium exhibits many notable signals that can be ascribed to a diverse array of low-molecular-mass biomolecules (Figure 1a, Figure 2a and Figure 3a). Certainly, ^1^H NMR resonances detectable included many metabolites, e.g., acetate, a number of short-chain organic acid anions, several nutrient substrates including carbohydrates (glucose, both α-and β-anomers), trace levels of vitamins (if present at detectable concentrations), N-acetylsugars (as their acetamido-NHCOCH_3_ functions), as well as amino acids such as glutamine, glutamate, glycine, lysine, arginine, proline, leucine, valine, histidine, tyrosine, phenylalanine and tryptophan, which are all vital for cellular growth. Also detectable was pyroglutamate, a product arising from the spontaneous decomposition of glutamine, which has been observed previously [26,27]. A full list of essential biomolecules presents the RPMI 1640 medium employed for our studies, as provided by the manufacturer, is available in Table S1 of the Supplementary Information Section.

In addition, a corresponding list of the all-metabolite resonance assignments, in addition to their related chemical shift values and coupling patterns, is presented in Table 1. For the spectra displayed, there were 53 signal resonances, 43 of which have been assigned to metabolites, although 3 of these remain tentative.

Particularly notable is the ^1^H NMR detection of pyroglutamate (5-oxo-pyrrolidine-2-carboxylate or 5-oxoproline), a cyclic lactam degradation product of glutamine, which, along with ammonia (also from this source), has been previously shown to exert negative effects regarding the culture of cells, and their maintenance and management [27]. From our quantitative ^1^H NMR data, it was estimated that the ratio of the 14C:14B signal intensities was approximately 1:1, but this would not represent a [pyroglutamate]:[glutamine] ratio of *ca*. 2:1, since the 14C pyroglutamate signal unfortunately superimposes somewhat on that of hydroxyproline’s 1/2-β-CH_2_ group (*ddd*). Therefore, it appears that a significant proportion of the glutamine originally added (expected concentration 2.055 mmol/L, Table S1) was degraded to pyroglutamate since the time that it was added to the culture medium formulation by the manufacturer and purchased by us. Equilibration of RPMI 1640 culture medium samples with progressively increasing supplemented concentrations of paramagnetic Ni(II) and Cu(II) ions resulted in alterations to the normalised intensities and/or linewidth broadenings of selected biomolecule resonances in a concentration-dependent manner, as evidenced from their ^1^H NMR analysis. Details of these modifications are provided below in Section 2.1 and Section 2.2 below.

### 2.1. Ni(II) Spectral Titration Experiment Results

In the high-field spectral region, selective broadening and corresponding reductions in the intensities of a series of ^1^H NMR resonances were observed, and these included those of glutamine, acetate, methionine, lysine, threonine and BCAAs (Figure 1). Moreover, higher levels of added Ni(II) resulted in further broadenings, e.g., that of aspartate’s 2.68 ppm resonance; interestingly, two new signals appear in these high-field profiles at an added Ni(II) level of 140 µmol/L (Figure 1b). However, overall one-way ANOVA analysis of the high- and medium-field regions of spectra found that significant Ni(II)-induced changes in the spectral profiles were in the sequential order: methionine > hydroxyproline > aspartate ≈ asparagine > leucine ≈ lysine ≈ α-glucose > glutamine > proline > N-acetyl transfer agents > acetate ≈ arginine > β-glucose > methanol for the 0.80–5.40 ppm regions of spectra. The effect of added Ni(II) on the acetate-CH_3_, and threonine and methionine side-chain-CH_3_ group signals (δ = 1.915 (*s*), 1.337 (*d*) and 2.141 ppm (*s*), respectively), was readily discernable, indicative of their involvement in the complexation of this transition metal ion in the culture medium evaluated (these changes are exhibited by purple arrows in Figure 2c).

Further broadenings of all of the above signals were readily visible at an added Ni(II) concentration of 540 µmol/L (Figure 2d). Those detectable were accompanied by decreases in the overall intensities of the glucose resonances in spectra (notably via the α-Glucose-C1H signal at δ = 5.237 ppm). Methyl group signals, including those of leucine (signals 1 and 2, δ = 0.965 and 0.990 ppm, respectively), valine (signals 2 and 4, δ = 0.990 and 1.041 ppm, respectively), isoleucine (signals 2 and 3, δ = 0.990 and 1.022 ppm respectively), together with the glutamine-γ-CH_2_ multiplet (signal 14B, δ = 2.433 ppm), all showed steady decreases in intensity, an observation also clearly visible in Figure 2.

Further data obtained in this titration experiment featured the aromatic regions (δ = 6.50–7.80 ppm) of ^1^H NMR spectra acquired (Figure 3), which exhibited clear and distinct resonances attributable to the amino acids tyrosine, histidine and phenylalanine present in the untreated culture medium. Upon the addition of Ni(II) at a concentration of only 71 µmol/L, evidence for its complexation by the amino acid histidine was obtained, and this was revealed by its two imidazole ring proton singlet signals undergoing a selective broadening process, which eventually culminated in their total removal from the spectrum following the subsequent addition of higher added Ni(II) concentrations, as depicted by the orange arrows in Figure 3c. This elimination of histidine’s -C4H and -C2H imidazole ring proton resonances is indicative of the significantly potent chelating capacity of Ni(II) by this particular amino acid [13]. Also notable at the added Ni(II) level of 140 µmol/L were significant decreases in the intensity of the tyrosine aromatic rings. Similarly, at this concentration, both the aromatic signals of tryptophan disappeared from the spectrum (Figure 3). Hence, overall, the biomolecules most influenced by added Ni(II) ions were found to be histidine > glutamine > acetate ≈ methionine ≈ lysine ≈ threonine ≈ BCAAs > tyrosine ≈ tryptophan > aspartate ≈ asparagine.

### 2.2. Cu(II) Spectral Titration Experimental Results

**Figure 4:**
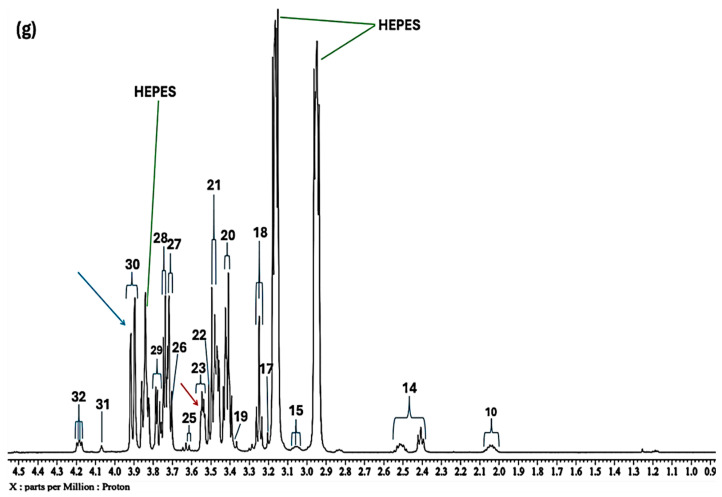

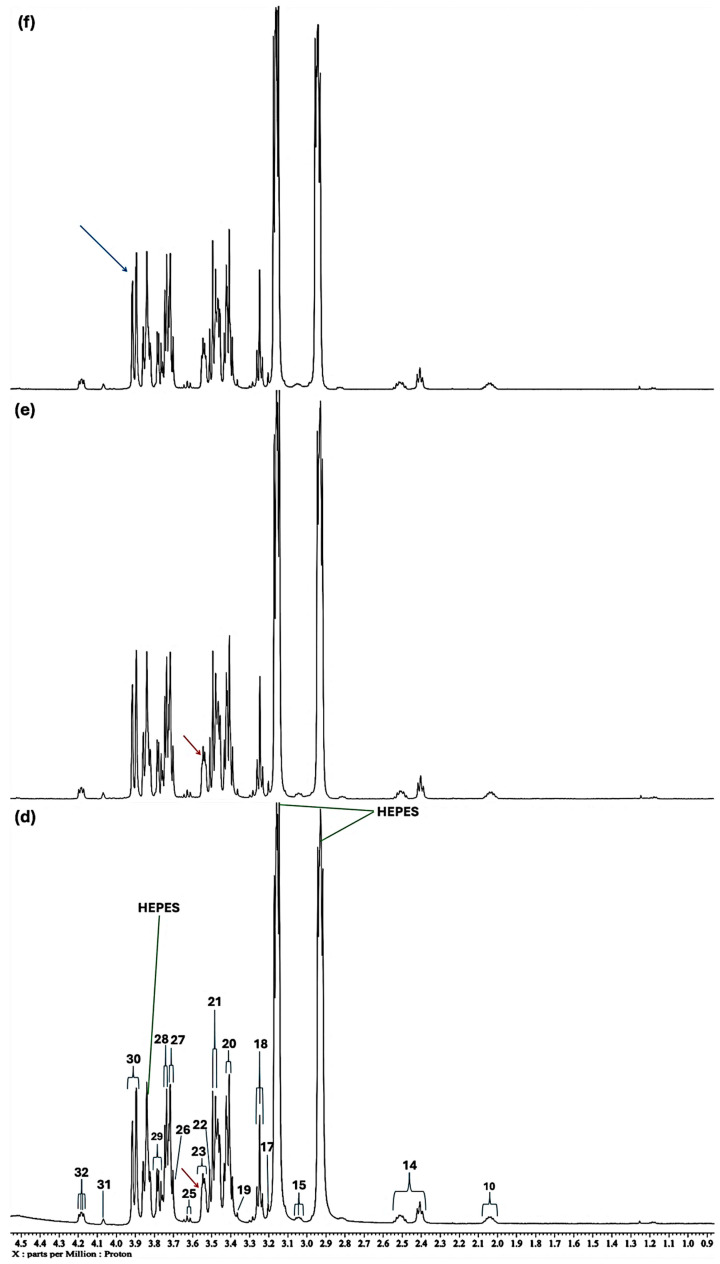

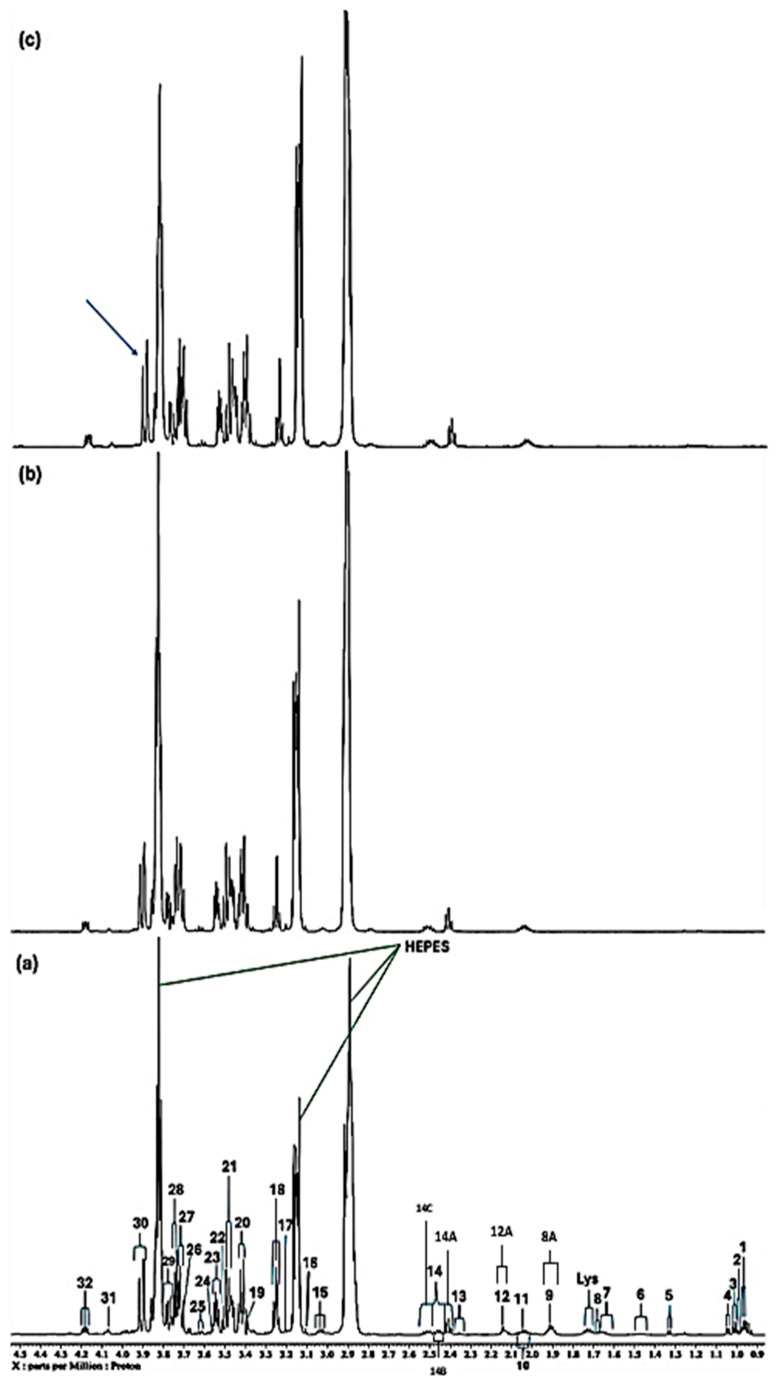
High- and medium-field (0.86–4.54 ppm) regions of the 600.17 MHz WASTED II ^1^H NMR spectra of (**a**) a control RPMI 1640 culture medium sample (untreated sample) devoid of any added Cu(II) ions, and the same sample following equilibration with Cu(II) at added final concentrations of (**b**) 7.1, (**c**) 14, (**d**) 28, (**e**) 41, (**f**) 54 and (**g**) 67 µmol/L. Typical spectra are shown. Assignment abbreviations: as Figure 1. Green lines represent the three large NMR-distinctive resonances of the HEPES buffer system (HEPES represents 4-(2-hydroxyethyl)-1-piperazine ethanesulphonic acid). The red and blue arrows in spectra refer to signals 23 and 30, i.e., α-Glucose-C2H and β-Glucose-C6H_a_ resonances, respectively.

**Figure 5:**
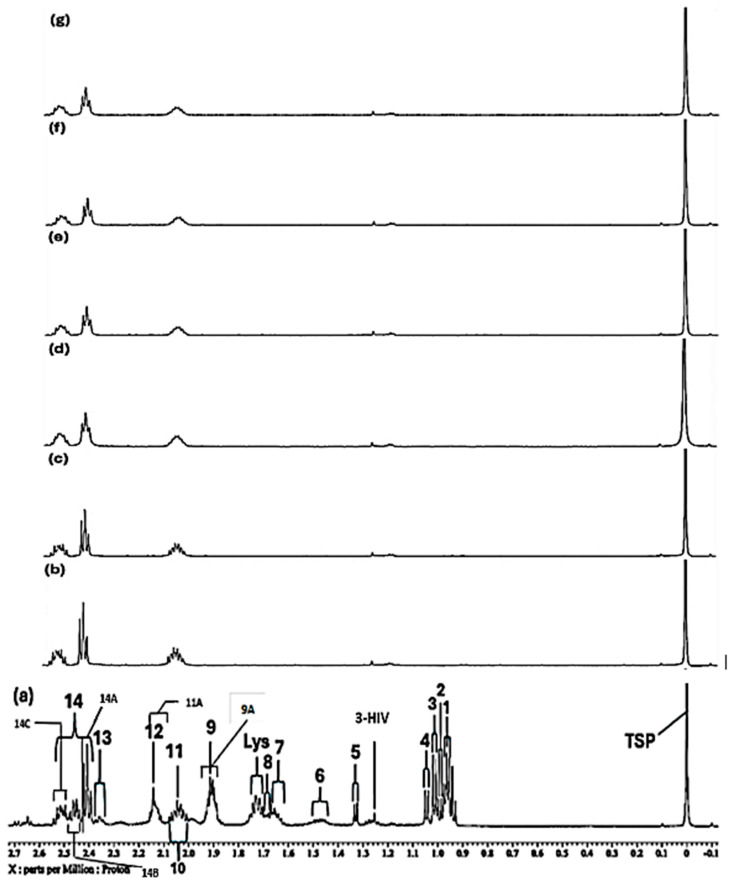
Expanded −0.10–2.56 ppm regions of the 600.17 MHz WASTED II ^1^H NMR spectra of (**a**) a control RPMI 1640 culture medium sample (untreated sample) devoid of any Cu(II) ions, and the same sample following equilibration with Cu(II) at added final concentrations of (**b**) 7.1 µmol/L, (**c**) 14 µmol/L, (**d**) 28 µmol/L, (**e**) 41 µmol/L, (**f**) 54 and (**g**) 67 µmol/L. Typical spectra are shown. Assignment abbreviations: As Figure 1 and Figure 2.

Similarly, the low- and high-frequency regions of ^1^H NMR spectra obtained from a typical titration involving the addition of 0–67 µmol/L Cu(II) to the RPMI 1640 culture medium are represented in Figure 4 and Figure 5, and 6 respectively (all Cu(II) ion-mediated spectral changes noted in Figure 4 are more clearly observed in their corresponding expanded spectral regions shown in Figure 5). These resonance modifications are also listed in Table 1, which shows results from the one-way ANOVA analysis of sequential 0.04 ppm buckets of the spectral profiles.

During the first series of NMR-based titrations, which involved added Cu(II) concentrations equivalent to those of Ni(II) above (71–670 µmol/L), virtually all resonances present in the ^1^H NMR profiles of the culture medium broadened beyond visualisation and detection at the lowest level equilibrated (71 µmol/L). Therefore, the stock titration solution of Cu(II) was diluted both 10- and 100-fold so that their final concentrations were 1.00 and 0.10 mmol/L, and these titrations were then repeated. Figure 4 shows the high-field region of spectra of the culture medium acquired prior to and following the addition of final levels of 7.1, 14, 28, 41, 54 and 67 µmol/L Cu(II). Clearly, at the lowest added level, a marked broadening of virtually all signals was observed, the most prominent being decreasing intensities of the glutamine- and glutamate-γ-methylene group multiplet signals, and those of all three BCAAs, along with nearly all further amino acids present, e.g., lysine, arginine and proline, etc. Furthermore, at this Cu(II) level, the only signals in the high-field region not affected by added Cu(II) were proline and hydroxyproline, although they did eventually broaden at an added concentration of 28 µmol/L. However, as we might expect, resonances ascribable to the two glucose anomers appeared to be largely unaffected by increasing concentrations of added of Cu(II) ion, although that of the β-Glucose-C6H_a_ proton (signal 30) was diminished in intensity somewhat when expressed relative to those of the myriad of other glucose resonances (Figure 4), and this indicates that the -CH_2_OH function at this position in the glucose ring (β-anomer) may play a minor role in added Cu(II) ion complexation. Interestingly, at an added Cu(II) concentration of 14 µmol/L, new minor signals were generated in the spectral profile at δ = 0.88 and 1.39 ppm (both doublets). This observation is not simply explicable, although the generation of agents responsible for such resonances may indeed arise from the Cu(II) ion-mediated oxidation of culture medium biomolecules. Completely randomised design ANOVA of the CSN-based dataset gave a significance ranking order of methionine > pyroglutamate > aspartate > leucine/lysine > asparagine > α-glucose > glutamine ≈ unknown > N-acetylsugars > glutamate/proline > acetate ≈ arginine for the high- and medium-field regions of spectra only.

At higher concentrations of added Cu(II) (≥28 µmol/L), the prominent -CH_2_OH ^1^H NMR signal of the HEPES buffer system (*δ* = 3.85 ppm) was significantly reduced in intensity, and this could potentially be ascribable to the ability of this function to coordinate to this metal ion, a process involving a fast exchange rate of it at its complexation centre. This effect was first noted at an added Cu(II) level of 14 µmol/L and continued sequentially with increasing concentrations. However, with Ni(II) it appeared that both the δ = 2.88 and 3.85 ppm HEPES buffer resonances were affected by added Ni(II), and this indicated that both the heterocyclic ring N-donor and -CH_2_OH functions play roles in complexing Ni(II).

**Figure 6:**
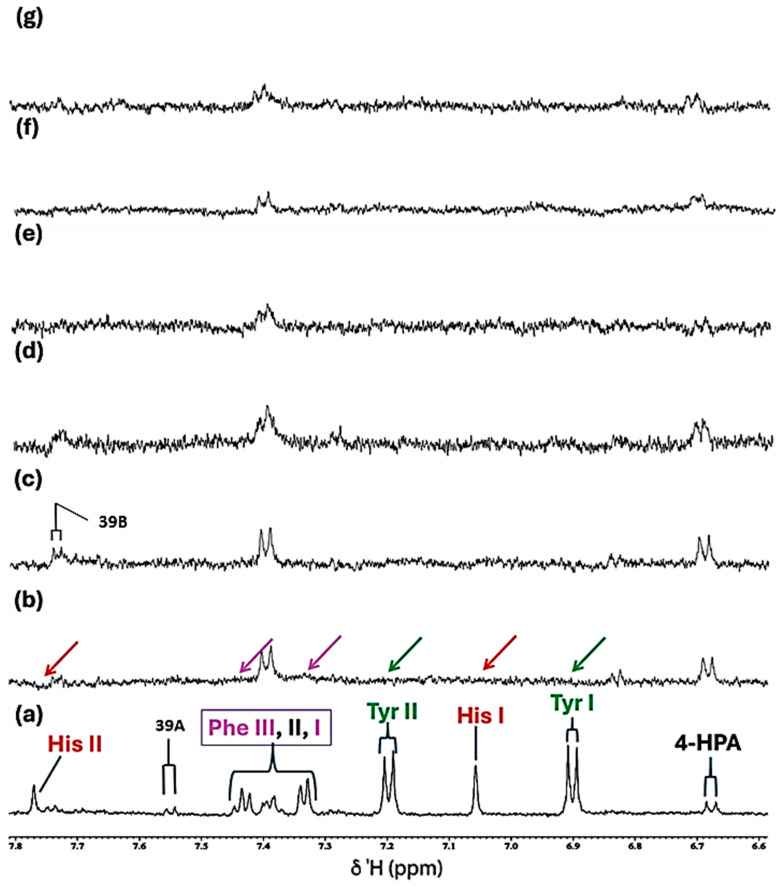
Partial 5.06–7.82 ppm regions of the 600.17 MHz WASTED II ^1^H NMR spectra of (**a**) a control RPMI 1640 culture medium sample (untreated sample) devoid of any added Cu(II) ions, and the same sample following equilibration with Cu(II) at added final concentrations of (**b**) 7.1, (**c**) 14, (**d**) 28, (**e**) 41, (**f**) 54 and (**g**) 67 µmol/L. Typical spectra are shown. Assignment abbreviations: As Figure 3. The green, red and purple arrows represent complete broadenings of the tyrosine, histidine and phenylalanine resonances following the addition of only 7.1 µmol/L Cu(II).

For the aromatic region of these spectra (δ = 6.50–7.80 ppm), which contained traces of what appears to be 4-hydroxyphenylacetate, and which is presumably a minor contaminant in the culture medium explored, along with the distinctive tyrosine, histidine and phenylalanine resonances (Figure 6a), addition of Cu(II) at a concentration of only 7.1 µmol/L, gave spectra which were consistent with the complexation of this metal ion by the amino acid histidine. This was evidenced by the disappearance of its two imidazole ring singlet signals (as observed with Ni(II)), i.e., they underwent selective and substantial broadening, resulting in their total removal at this added Cu(II) level. Also notable at higher added Cu(II) levels (≥14 µmol/L) was a significant broadening of the tyrosine and phenylalanine aromatic ring resonances. Hence, the selective removal of these aromatic ring proton resonances is indicative of the potent complexing capacity of Cu(II) demonstrated by each of these amino acids, although histidine is clearly the most powerful ligand.

In view of these results, we elected to use a further diluted stock solution of Cu(II) which engendered final culture medium addition levels of only 0.71 to 6.70 µmol/L. However, with the exception of the highest level added (6.7 µmol/L), where some significant broadening of the histidine imidazole ring proton resonances was detected, all lower concentrations of Cu(II) added did not affect any resonances throughout the entire spectral profile range.

Notably, however, we observed that the TSP internal standard -Si(CH_3_)_3_ resonance broadened with added Cu(II) in a concentration-dependent manner, as outlined in Section S3 of the Supplementary Information. Indeed, significant broadening of this signal commenced at an added level of only 7.1 µmol/L. However, despite a significant level of broadening, it appeared that all of the resonance was encapsulated within the −0.20 to 0.20 ppm 0.40 ppm width bucket region utilised for its intensity determination, and therefore it was tentatively employed as a denominator for the determination of culture medium metabolites, as described in Section 2.3. This broadening effect was also observed for added Ni(II), albeit at much higher added levels, i.e., most especially at concentrations ≥140 µmol/L, as previously described in Ref. [13]. For these reasons, TSP was only used as a semi-quantitative internal standard for this study, and TSP-normalised datasets are always shown in conjunction with those which were CSN for comparative purposes.

Additionally, the concentration of H^+^ ion levels (pH value) remained stable before (untreated control) and after the addition of rising added concentrations of Ni(II) and Cu(II) into the RPMI 1640 culture medium, since chemical shift values were coherent throughout, and this was attributed to the enhanced buffering capacity of the HEPES system present in the culture medium employed, presumably also with significant contributions from bicarbonate/carbonate and the phosphate systems incorporated (Table S1). Indeed, pH monitoring using a hand-held pH metre confirmed this.

Although the chemical nature of the precipitate formed on addition of Cu(II) to the culture medium is unclear, it should be noted that insoluble Cu(OH)_2_ is only generated in the 8.5–9.5 pH range, and therefore it cannot be formed at pH values at or close to neutrality as conducted in the current study. Furthermore, the black-coloured copper(II) oxide (Cu(II)O) species arises from Cu(OH)_2_ decomposition at temperatures as high as ca. 100 °C, and hence this oxide also cannot constitute this precipitate. However, it is important to note that the redox reaction of added Cu(II) with D-glucose yields insoluble, brown/red-coloured Cu(I)_2_O, which has been detected in further studies involving the interaction of this metal ion with glucose-containing culture media [30]. This process is further discussed in the Discussion Section (Section 3) of this paper.

Nonetheless, selected resonance intensities may diminish simply because their assigned metabolites precipitate out of solution, perhaps as constituents of copper(II)-phosphate and/or -carbonate (malachite-type) materials possibly as stabilising encapsulating agents for any nanoparticulate species formed. Therefore, the levels of amino acids and further biomolecules remaining in solution are those which endure and respond to ^1^H NMR analysis, and it is conceivable that these levels may be significantly lower than those present in the original culture medium in view of co-deposition processes.

### 2.3. ^1^H NMR-Based Metabolomics Analysis of Ni(II) and Cu(II) Spectral Titration Datasets

#### 2.3.1. ANOVA Results

One-way completely randomised design ANOVA was applied to determine the statistical significance of the replicate titrations performed with either added Ni(II) (71–670 µmol/L), or a 10-fold lower concentration range of Cu(II), and *p* values for the statistical significance of each ^1^H NMR bucket within the ^1^H NMR spectral profile range of 0.90–5.40 ppm, are reported in Figure 1 for the CSN-normalised dataset. These indicate the most important metal ion-mediated changes to resonances, and the orders of these for both metal ions added are provided in Section 2.1 and Section 2.2 above. However, it was clear that for the aliphatic biomolecules explored here, glutamine, aspartate, acetate, methionine and lysine and BCAAs were important culture medium complexants for both these metal ions.

For experiments involving Cu(II) titrations. some typical decreases in resonance intensities for the 0.96–1.00, 1.00–1.04, 1.04–1.08, 1.68–1.72, 1.72–1.76 and 1.92–1.96 ppm buckets are viewable in the violin plots shown in Figure S1 (Supplementary Information). For all of these, the resonance intensity decreases to zero following the very first addition of Cu(II). However, it should also be noted that since the CSN normalisation fixed 0.04 ppm ^1^H NMR bucket approach was used, in cases where the resonances were only partially or not completely extinguished following the first or subsequent additions of Cu(II) (7.1 up to a final level of 67 µmol/L), these signals actually increase rather than decrease in normalised intensity, because their contribution towards a 100% spectral profile observed at each increasing added Cu(II) concentration was actually sequentially greater rather than lower following the Cu(II) ion-induced disappearance of quite a large number of other spectral resonances at these respective added levels; examples of this can be viewed for the 3.20–3.24 and 3.80–3.84 ppm glucose resonance buckets shown in Figure S1. Similar problems were encountered when using median or product quotient normalisation (PQN) strategies for normalisation purposes. This phenomenon, and explanations offered for it, is discussed in more detail in Ref. [13].

**Figure 7:**
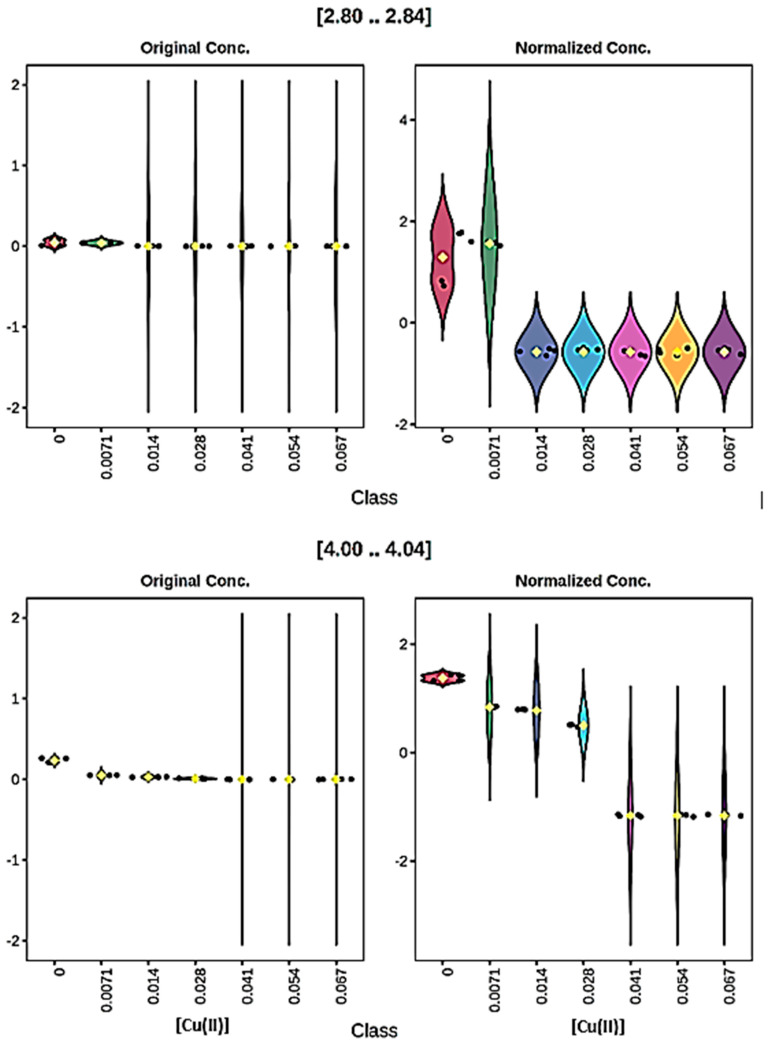
Plots of ‘raw’ (**left-hand-side**) and normalised (**right-hand-side**) ^1^H NMR signal intensities ([2.80..2.84] (**top**) and [4.00..4.04] (**bottom**), 2.80–4.00 and 4.00–4.04 ppm buckets respectively) for the RPMI 1640 culture medium titrated with increasing concentrations of Cu(II). These data show examples of added ‘concentration lags’ which occur in the ranges of 0–0.0071 and 0–0.028 mmol/L, respectively, for these buckets. Datasets were constant sum-normalised (CSN), glog_10_-transformed and Pareto-scaled prior to analysis. Different colours correspond to different added Cu(II) concentrations.

With this CSN normalisation process, for the Cu(II) titration there was a total of 23 out of 71 buckets which featured statistically significant decreases in resonance intensities. Although most of these involved immediate 100% reductions following addition of the lowest added level of this metal ion (7.1 µmol/L only), a small number of those observed involved a concentration-dependent lag of up to 28 µmol/L before observing decreases in intensities. Two examples of these observations (i.e., those of the 2.80–2.84 and 4.00–4.04 ppm buckets) are provided in Figure 7. Therefore, overall these results provide evidence for a series of different classes of Cu(II)-responsive amino acids and other biomolecules, with some of them scavenging it at its lowest added level, others not responding intensity-wise until larger amounts were added, with yet others being quite resistant to complexation, and hence resonance broadening, until the highest added concentration had been attained.

For the Ni(II) titrations conducted (71–670 µmol/L), using the CSN normalisation approach, again there were differing types of concentration-dependent resonance responses towards this added metal ion, with both decreases and increases in intensities being observed. Indeed, of the many buckets which revealed statistically significant changes, there were 17 downregulations in intensity, of which approximately one-half involved a concentration-dependent lag phase. Moreover, there were 18 resonance intensity increases, of which several featured significant concentration lags.

As noted in Section S3, there were some significant concentration-dependent added Cu(II)-induced broadenings of the TSP internal standard reference signal observed (Figures S2 and S3). However, it should be noted from Figure S2 that despite the Δv_1/2_ value of this resonance more than doubling on enhancing the added Cu(II) concentration from zero to 67 µmol/L, all of the signal remained within the −0.20 to +0.20 ppm bucket region, which was employed for its electronic integration. Therefore, assuming that none of this signal was hyper-shifted to extreme chemical shift values, we elected to perform internal TSP normalisation of biomolecular bucket signals prior to glog_10_-transformation and Pareto-scaling. The top 30 signals resulting from experiments involving Ni(II) and Cu(II) ion titrations are listed in Tables S2 and S3. These data indicate that the culture medium metabolites most affected by added Ni(II) ion were Arginine > Lysine > Glutamate ≈ Proline ≈ Pyroglutamate > Glutamine > Leucine > Asparagine, whereas those most influenced by added Cu(II) were all BCAAs > Asparagine > Acetate > Lysine > Methionine > Glucose, the latter presumably because of its high culture medium concentration.

#### 2.3.2. Significance Analysis of Metabolomics (SAMs) Involving ^1^H NMR Bucket Variables

For the Cu(II) titration data (featuring added levels of 0–67 µmol/L), this MV analysis yielded very low, very highly significant *q* values (i.e., very close to zero), and the rank Cu(II)-broadening order of these found was found to be isoleucine > β-glucose > lysine > acetate > valine > methionine > leucine > pyroglutamate > arginine > dimethylsulphone > threonine > methanol > aspartate > N-acetylsugars (q ≈ 0 for all of these), and cystine (*q* = 0.002); a *q* value is equivalent to an FDR-corrected *p* value. These selections are also in quite good agreement with those determined by ANOVA. Likewise, for the Ni(II) titration dataset (added levels 0–670 µmol/L), again very low *q* values were achieved, the rank Ni(II)-broadening order being aspartate > β-glucose > pyroglutamate > asparagine > cystine > acetate > methionine > pyroglutamate/hydroxyproline > lysine > valine > methanol > leucine (*q* ≈ 0 for all of these). More than 40 bucket variables were statistically significant for both analyses.

#### 2.3.3. Random Forest (RF) Analysis

Finally, the RF strategy, an AI-based machine-learning MV technique, was employed for determining differences between the control culture medium sample replicates and each of those treated with increasing added concentrations of Ni(II) or Cu(II). This model was very successful in distinguishing between the ^1^H NMR patterns of each sample for both these metal ions. Indeed, Table 2 shows that for a total of n = 4 replicates per titration point, there is an excellent (100%) level of discrimination between each of the sample groups which were treated with either 0, 71, 140, 280, 410, 540 and 670 µmol/L Ni(II), or 0, 7.1, 14, 28, 54 and 67 µmol/L Cu(II).

### 2.4. ATR-FTIR Analysis of Deposit Formed from Addition of Cu(II) to RPMI 1640 Culture Medium

As noted in Section 2, the pale blue deposit formed on addition of Cu(II) to the culture medium will be expected to significantly deplete this metal ion in this aqueous matrix, perhaps in an added concentration-dependent manner. Indeed, this has been noted by other researchers working on the ‘spiking’ of this metal ion into different types of culture medium for the growth of bacteria [30].

Moreover, selected biomolecular complexants may also be removed from this solution titration medium, most especially if any of them are associated with the deposited material, perhaps as encapsulating agents surrounding an inorganic core such as copper(II)-phosphates or -carbonate, and perhaps also in nanoparticulate-type materials. Therefore, we attempted to explore the chemical nature of this material, which may comprise a copper(II)-phosphate- and/or -carbonate-based species, perhaps with associated solid matrix amino acid metabolites provided by the medium involved.

Despite some quite clear similarities, ATR-FTIR spectra acquired on the growth culture medium showed some differences between the Cu(II)-induced solid matrix produced and that of untreated culture medium (Figure S4). However, in these spectra, water vibrational bands located at ca. 3290 cm^−1^ (O-H stretching), 1634 (H-O-H bending), and 580 and 520 cm^−1^, prevailed [31]. Major absorption bands arising from glucose (initial culture medium concentration 11.1 mmol/L) in the 1000–1200 cm^−1^ range (C–O–C stretch, C–OH stretch, C-OH and C-O-C deformations, and pyranose ring vibrations) were visible in the ATR-FTIR spectrum of the control culture medium and its supernatant following removal of the deposit (these bands are much sharper than the broader water bands observed) [31]. Hence, on consideration of these data, and those available in Section S4, the ATR-FTIR data acquired indicate that the deposit isolated from Cu(II)-culture medium admixtures may contain amino acid species associated with insoluble Cu^II^PO_4_^3−^ and/or -CO_3_^2−^ inorganic adducts. However, further investigations are clearly required to characterise the chemical nature of this matrix.

### 2.5. Application of EDTA to Reverse Cu(II)-Induced ^1^H NMR Resonance Broadenings

**Figure 8:**
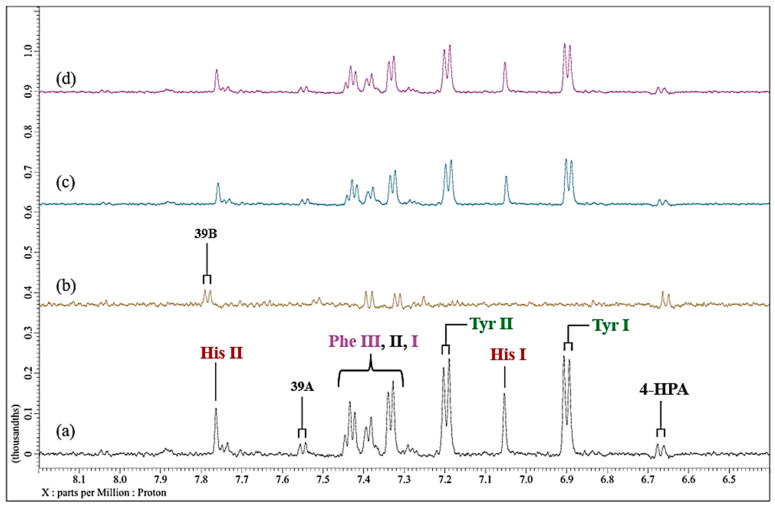
Effect of added EDTA on the ^1^H NMR profiles of Cu(II)-loaded RPMI 1640 culture medium. Partial (expanded) 6.40–8.20 regions of 600 MHz WASTED ^1^H NMR profiles of (**a**) untreated culture medium; (**b**) as (**a**), but spiked with 6.0 µmol/L Cu(II)_(aq_._)_; (**c**), as (**b**), but subsequently treated with a 10-fold excess (60 µmol/L) of the metal ion chelator EDTA (spectrum acquired 30 min following addition); (**d**), as (**c**) but spectrum acquired 24 h following EDTA treatment. Typical spectra are shown. Abbreviations: as Figure 3 and Figure 6.

Since the above Cu(II)-induced spectroscopic modifications observed above should be reversible, assuming that sufficient amounts of this metal ion remain in the culture medium, we subsequently added the powerful hexadentate chelator EDTA to Cu(II)-treated solution following removal of any deposit formed initially. For this purpose, we added a final concentration of 6.0 µmol/L Cu(II) to the culture medium and again observed a marked broadening of the imidazole ring protons of L-histidine (Figure 8). However, following the addition of a ca. 10-fold excess of EDTA to that of added Cu(II), the histidine resonances reappeared to the spectral profiles, which were acquired at time-points of 0.5 and 24.0 h post-EDTA addition (no time-dependence of the intensities of these signals was noted).

However, these regenerated signals certainly appeared to be significantly broader than those of the untreated culture medium, indicating that at least some Cu(II) remains coordinated to this amino acid. Additionally, resonances of tyrosine and, to a lesser extent, phenylalanine, which again were previously substantially broadened by added Cu(II), reappeared in the spectra acquired at these time-points. Moreover, those of a series of aliphatic biomolecules (e.g., glutamine, BCAAs, etc.) were also found to return to ^1^H NMR spectra of Cu(II)-treated culture medium. Of course, this exchange process is very thermodynamically favourable, with a high stoichiometric, thermodynamic equilibrium constant (*K*_eq_), i.e., this experiment confirms EDTA’s powerful Cu(II) ion-chelating ability.

As expected, subsequent to excess EDTA addition, a resonance ascribable to its 1:1 Ca^2+^ complex (ethylenic proton singlet at δ = 2.57, and -NCH_2_CO_2_^−^ proton AB coupling pattern signal at δ = 3.06–3.17 ppm) were readily visible in the spectra acquired, along with those of ‘free’ (uncomplexed) EDTA at ca. 3.2 and 3.6 ppm respectively.

**Figure 9:**
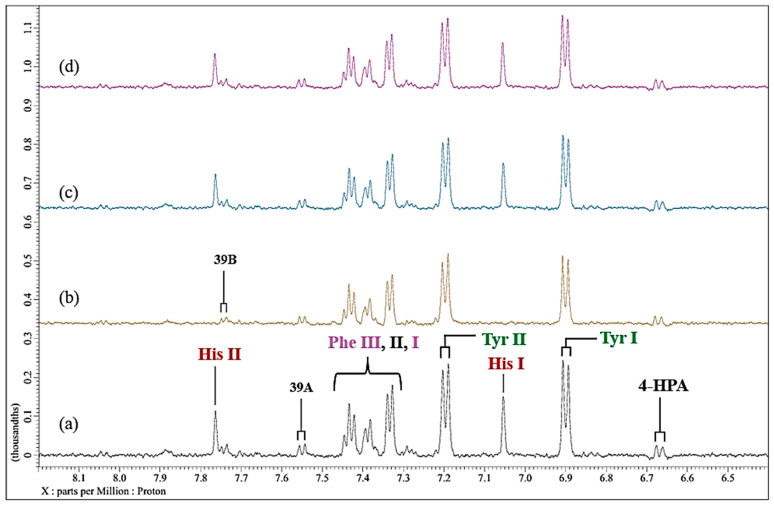
Influence of added EDTA on the ^1^H NMR profiles of Ni(II)-loaded RPMI 1640 culture medium. Expanded) 6.40–8.20 regions of 600 MHz WASTED ^1^H NMR profiles of (**a**) untreated culture medium; (**b**) as (**a**), but spiked with 60 µmol/L Ni(II)_(aq_._)_; (**c**), as (**b**), but subsequently treated with 1.00 molar equivalent (60 µmol/L) of EDTA (spectrum acquired 30 min following addition); (**d**), as (**c**) but after acquiring the spectrum 24 h subsequent to EDTA treatment. Typical spectra are shown. Abbreviations: as Figure 3, Figure 6 and Figure 8.

These experiments involving EDTA were also conducted with Ni(II), although in this case a final level of 60 µmol/L was added to the culture medium, which was sufficient to completely broaden the histidine imidazole ring proton resonances in the aromatic regions of spectra acquired (Figure 9a,b). However, only one molar equivalent of added EDTA was required to return these ^1^H NMR signals close to their original status (Figure 9). There appeared to be little or no time dependence of this process. Also notable was that the ^1^H NMR resonances of other aromatic amino acids, e.g., those of phenylalanine and tyrosine, were affected much less so than that of histidine, and this observation reflects the magnitudes of the thermodynamic stability constants of these amino acid complexes with Ni(II) [13].

## 3. Discussion

This study was explicitly focused on the identification of any stable complexes produced from selective reactions between added low-molecular-mass metal ions (Ni(II)_(aq_._)_ and Cu(II)_(aq_._)_) and biomolecular complexants of the RPMI 1640 culture medium. These processes potentially exert major influences on the cytotoxicities, bioavailabilities and biodistribution of such metal ions when tested, or toxicologically evaluated, in cell culture experiments. As noted above, these trace metal ions, notably those of Cu(II), have been documented to facilitate the production of ROS in the presence of intra- and extracellular reducing agents, a process coupled with the impact of their speciation status on their thermodynamic redox potential parameters [19,20,21,32,33,34]. Cautious ^1^H NMR spectral titrations of this culture medium with increasing, albeit trace concentrations of Ni(II) and Cu(II), provided a high level of scientific evidence for their complexation, and its rank order, by biomolecules present in this medium (Table 1). Notably, the paramagnetism of such complexed metal ions substantially diminishes the relaxation times (both T1 and T2) of co-ordinated ligands; although this phenomenon reduces spectral acquisition times and data collection rates, it can also give rise to complexation- and exchange-dependent broadened signals, most especially if T2 values are significantly diminished.

High-resolution NMR analysis enables the detection of changes in resonance frequencies and spin-system profiles resulting from the formation of complexes, as well as entailing the proficiency of this process to permit concurrent assessments of the Cu(II) and Ni(II)-complexing capacities of an extensive range of culture medium biomolecules simultaneously, and in a practically non-destructive manner, leaving the samples available intact for further analysis, if required. Other advantages include less essential sample preparation, outstanding reproducibility, and a high level of intra-sample and inter-assay precision. Moreover, a single internal standard is often satisfactory for complete quantitation of all metabolites detected in the spectrum, computerised results procurement, and the identification of molecules (unknown metabolites) is achieved via comparisons with now available extensive libraries of NMR spectra, which was previously a key ‘bottleneck’ in metabolomics research activities [35]. Notably, NMR methods available for the speciation of metal ions continue to advance for the precise detection and quantification of metabolite ligands involved in complex formation [36].

The biomolecular ^1^H NMR analytical data obtained in the current investigation have determined that specific amino acids, including L-histidine and L-glutamine, serve as pivotal nickel(II) and/or copper(II) ion-complexing agents present within the RPMI 1640 culture medium; whether acting independently or synergistically, these metabolites may substantially influence the biological activity and remodel the potential toxicological activities of these redox-active metal ions, including respiratory carcinogenicity [37] and neuronal dysfunctions such as Alzheimer’s diseases [38].

Both Ni(II) and Cu(II) exhibit a pronounced binding affinity towards harder (O)- and (N)-donor ligands, in addition to those which are softer (S)-donors. Hence, these metal ions may complex robustly to organic compounds comprising amino, carboxylato and thiol(ato) functions [17,39], equilibria resulting in the formation of metal ion complexes of varying geometries and stabilities. Specifically, Ni(II) ions can exist as two major structural forms which are related to their ^1^H NMR spectral profiles in solution. These are diamagnetic square planar complexes which, in the absence of rapid exchange-broadening on the NMR timescale, will not be expected to modify resonance linewidths or δ values significantly. However, paramagnetic tetrahedral, octahedral or alternative complexes of Ni(II) will, of course, have broader and paramagnetically shifted signals. Contrastingly, all Cu(II) complexes are paramagnetic, and therefore their resonance linewidth and δ values are substantially enhanced.

The ligand exchange rate for octahedral Ni(II) complexes is comparatively rapid (e.g., for Ni(II)(H_2_O)_6_^2+^ ion it is 3.2 × 10^4^ s^−1^ at 25 °C) [40]. In 2004, Carrera et al. [41] demonstrated that Cu(II) complexation by excess histidine produced a stable 1:2 complex of square planar geometry, with the side-chain imidazole-N performing a vital function in its stabilisation.

In the current study, at an added Ni(II) concentration of 71 µmol/L, complexation with histidine results in the broadening, and attenuation of the overall normalised intensities of its -C2H and -C4H imidazole ring ^1^H resonances, in contrast to results observed with tyrosine and phenylalanine aromatic ring proton signals in the δ = 6.50−7.80 ppm region (Figure 3), i.e., substitution-induced broadening is noted, whereas, in the case of the Cu(II) titration experiment, only minimal amounts of Cu(II) (≤7.1 µmol/L) were necessary to induce a selective and complete broadening of the histidine resonances (Figure 6). These results are in line with previous speciation experiments conducted with osteoarthritic knee-joint synovial fluid [24]. However, Williams and co-workers [42] performed an earlier computational investigation in which a hypothetical distribution of Cu(II) species was ascertained based on the stability constants of complexes of Cu(II) with amino acids and other metabolites present in human blood plasma; such distributions were also explored in other biofluids too. They found that both histidine and alanine were integrated into all predicted Cu(II)-amino acid complexes that are likely to exist in human blood plasma, most especially at low levels. Both observations underscore the favoured complexation of histidine by these trace metal ions, thereby highlighting the significant role that this amino acid performs in the homeostasis of both Ni(II) and Cu(II) within biological systems. Moreover, although Naughton et al. [24] found that in inflammatory knee-joint synovial fluid ultrafiltrate, added Cu(II) at levels of 14–72 µmol/L was predominantly complexed to histidine > alanine > formate > threonine > lactate > tyrosine > phenylalanine in that order, and therefore these complexation results are similar to those found in the current study. However, it should be noted that alanine was not present in the RPMI 1640 culture medium evaluated herein, and formate was only barely detectable (s, δ = 8.46 ppm) in the untreated spectral profiles acquired, and therefore it was not possible, nor strictly relevant, to assess their abilities to complex Ni(II) and Cu(II) ions in this investigation.

Previous investigations have also shown that there is an ‘exchangeable’ pool of Cu(II) ions which are is complexed to the terminal tripeptide site of albumin and amino acids in human blood serum [43,44]. Notably, it has been suggested that Cu(II)-amino acid complexes are of much physiological significance since they play an important role in the transport of this metal ion between blood and tissues [45,46]. Although a number of ternary Cu(II) complexes with histidine and further amino acids have been detected in human serum, at that point in time they had not been properly characterised [45]. Moreover, a series of computational simulations provided evidence that a ternary L-histidine-Cu(II)-L-glutamine complex is one of the most important low-molecular-mass Cu(II) coordination species in human biochemistry [46].In the case of the Cu(II) titrations performed in the current study, a further observation was the unexpected ‘appearance’ of minor doublet resonances ascribable to low-molecular-mass species appear in these high-field profiles at an added Ni(II) level of 140 µmol/L (Figure 2c). This phenomenon has been previously observed in other metal ion speciation and related studies, although such experiments have involved human biofluids, and therefore this effect has been ascribed to their mobilisation from a macromolecule-bound NMR-invisible “pool” of low-molecular-mass agents or metabolites to become NMR-visible “free” forms after adding an excess of selected reagents (including metal ions) to the biofluid matrix involved, usually those with the same electronic charge sign [47].

The trace metal ions evaluated here clearly form a range of metal ion-amino acid complexes exhibiting distinctive stoichiometries and protonation states. The resonance broadening effects observed are attributable to the powerful paramagnetic nature of these metal ions, particularly that of Cu(II), forming complexes with culture medium ligands, and their ability to exchange with them rapidly on the NMR timescale. Additionally, these amino acids have a superior binding affinity for copper(II) ions in order to create stable complexes, albeit those which rapidly exchange in the solution state. Once these complexes are formed, this markedly alters these biomolecules’ NMR-receptive ^1^H nucleus status (i.e., magnetic environment), resulting in the disappearance of their ^1^H NMR signals from the spectrum [48,49].

Of particular interest, the generation of these trace metal ion-histidinate complexes may be advantageous in diminishing the overall generation and release of ROS such as ^●^OH radical from these metal ion species, and hence also their toxic actions mediated by their high reactivities with ‘natural’ scavenging biomolecules. Both ‘free’ Ni(II)_(aq_._)_ and Cu(II)_(aq_._)_ ions are considered to be reactive as redox-active ions, and are recognised to disturb normal cellular processes, potentially by initiating “oxidative stress” through the adverse production of ROS, a process resulting in reduced cell viability, and eventually leading to apoptosis in the case of rapidly proliferating cancerous cells [20,22]. However, in vitro investigations have demonstrated that histidine exhibits powerful antioxidant properties, i.e., it acts as a successful ^●^OH radical scavenger (*k*_2_ = 3.0 × 10^9^ M^−1^ s^−1^ [50]), as do phenylalanine and tyrosine (*k*_2_ = 3.50 and 3.70 × 10^9^ M^−1^ s^−1,^ respectively [50]).

On reaction of Ni(II) ions with H_2_O_2_, L-histidine, glutathione or L-cysteine ligands effectively act as pro-oxidants and generate ^●^OH radical, but only when present at lower [ligand]:[Ni(II)] ratios (i.e., ≤1); at higher values, however, they serve as scavenging antioxidants [51]. These phenomena are explicable by the scavenging of any ^●^OH radical generated by either the free ligand (when present in excess), or alternatively ‘site-specifically’ by the Ni(II)-bound complexant when present as its *mono*- or *bis*-histidinato-complexes [52,53] (which, of course, are in equilibrium with the ‘free’ form of these ligands), and also by modifications in the redox potential of the Ni(III)/Ni(II) couple (*E*^0^) on complexation with these biomolecules.

Originally, the formation of Ni(III) complexes with a number of pyridine-oxime ligands in non-aqueous solution gave rise to qualitative criteria for ligands which stabilise this metal ion; moreover, a wide-ranging succession of tetra-aza macrocyclic nickel complexes in an acetonitrile solvent system has been employed to quantitatively correlate ligand natures with Ni(III):Ni(II) couple electrode potentials (*E*^0^s) [54]. Additionally, this early report provided values for the redox potentials of this couple for a series of 30 peptide complexes in an aqueous environment, and it was found that these values, which ranged from +0.96 to +0.79 V, decreased with the number of deprotonated peptide groups available. Interestingly, when present in these peptide ligands, histidine residues increased these values. Suggested ligand field stabilisations of the d^8^ *vs*. d^7^ or d^9^ electronic configurations in the square planar and tetragonal peptide complex domains provided an explanation for the smaller *E*^0^ values for Ni complexes than those observed for Cu [54].

It has been argued that such redox considerations are of critical importance regarding nickel toxicity. Intriguingly, these data indicate that ^●^OH radical generated from these ‘catalytic’ trace metal ions coordinated by histidine donor atoms might directly neutralise or remove this aggressively reactive radical species precisely where it is formed, i.e., via ‘site-specific’ hydroxylation processes indicated above. Moreover, the ^●^OH radical scavenging capacity of Ni(II)-complexed histidine is also likely to involve changes in the redox reactivity of these metal ions which promote ^●^OH radical production from H_2_O_2_ [55]. Hence, this process could mitigate oxidative damage inducible by Ni(II) and Cu(II) ions, both intra- and extracellularly. Of course, this pathway will significantly depend on H_2_O_2_’s ability to reach Ni(II)/Cu(II)-complexing locations, along with the specific amino acid involved in its speciation status. Earlier investigations have revealed that the interaction of H_2_O_2_ with Cu(II)-histidine complexes leads to the formation of asparagine, aspartate, aspartylurea, and formyl asparagine [24]. Furthermore, it has been established that Cu(II)-histidinate complexes demonstrate superoxide dismutase (SOD)-mimicking properties, a pivotal enzyme involved in the scavenging of ROS in vivo (SOD enzymes serve as a universal ‘shield’ against oxidative stress in view of their roles as catalysts in the dismutation of superoxide anion radicals (O_2_^●−^) to O_2_ and H_2_O_2_). The rate constant for the dismutation of O_2_^•−^ by Cu(II)-histidine complexes actually approximates that of SOD itself [56]. Interestingly, our ^1^H NMR data revealed that the amino acid asparagine significantly broadened on addition of Cu(II) as a ‘hard’ ligand/chelator class, albeit secondary complexant.

Nevertheless, complexation of Ni(II) and Cu(II) by histidine may have implications for their cellular uptake and delivery to their respective enzyme centres. For example, nickel(II) ion-dependent enzymes (e.g., urease and lactate racemase) in cells or microorganisms necessitate the employment of numerous accessory paths that promote the usage of this metal ion as key cofactors, while simultaneously mitigating harmful toxicological consequences. These enzymatic systems comprise transporters that control the import and export of Ni(II) ions through cell membranes, as well as intracellular Ni(II) ion-binding proteins that are pivotal for their distribution [57]. The ATP-binding cassette (ABC) transporter represents one of numerous classes of uptake mechanisms recognised as vital for the acquisition of Ni(II) ions in microbial organisms (e.g., *E. coli*), given that these transporters comprise a soluble binding protein that sequesters this Ni(II) substrate from the periplasmic space, and translocates it across the cellular transmembrane [58]. Ni(II)-histidinate complexes formed may potentially hinder recognition by these transporters, leading to a reduction or complete absence of Ni(II) uptake (i.e., a phenomenon affecting Ni(II) ion bioavailability), thereby adversely impacting cells that require it, such as *E. coli*, and subsequently obstructing critical enzymatic processes. Furthermore, despite its significant role, Ni(II) ions are typically inadequate when present at low nmol/L levels in Ni(II)-dependent bioenvironments [59], whilst significant quantities are required for the catalytic activities of Ni(II)-dependent enzymes. Therefore, adequate acquisition of Ni(II) ions by these enzymes constitutes a process of paramount importance, and which may be regulated by their speciation status.

However, glucose, which acts as the chief source of energy for numerous types of cells in culture, and also has the potential to influence cellular proliferation [60], was only marginally affected by the addition of these metal ions, albeit at their higher added levels, and therefore their complexation by this essential biomolecule, and perhaps other carbohydrates available, is unlikely to occur when they are present at trace levels in vivo. However, it may take part in redox reactions with added glucose to form insoluble brown/red Cu(I) oxide and gluconate [30].

L-glutamine is an essential amino acid which is vital since it acts as a secondary source of energy within cellular metabolic pathways and also offers nitrogen for NAD^+^/NADPH^+^-associated reactions [61]. When acting as a complexant for Cu(II), the metal ion is coordinated via the nitrogen and oxygen donor atoms of its amino and carboxylate functions, respectively, thereby facilitating the formation of a stable (Cu^II^(Gln)_2_) complex. Of course, this coordination process is imperative for maintaining its biological actions [62]. In inter-related investigations [63,64], Cu(II) complexes frequently demonstrate square pyramidal or octahedral configuration-distorted geometries, based upon the environment of the ligand. For example, Patra, Roy and Chakravarty [65], and Deschamps et al. [66], reported that the interaction between glutamine and Cu(II) ions within biological systems gives rise to the formation of various types of metal ion complexes.

In the current study, glutamine’s ^1^H NMR resonances displayed an added Ni(II) ion-dependent decrease in intensity with increasing added concentration. The same observation was made with added Cu(II), although sensitivity of this biomolecular ligand (as monitored by its γ-CH_2_ group multiplet signal (*δ* = 2.433 ppm, Figure 4 and Figure 5) was much greater than that observed with Ni(II), with a 10-fold decrease in Cu(II) concentration (7.1–67 µmol/L range) exerting approximately the same effect on the linewidth of this and other complexing metabolite signals as that of 71–670 µmol/L Ni(II). Therefore, these results are attributable to the formation of a Cu(II)-glutamine complex featuring a coordination geometry potentially containing several sites that may serve to challenge the fast ligand exchange phenomenon. An electron paramagnetic resonance (EPR) study reported that in the solid-state, the coordination of Cu(II) with glutamine involves two nitrogen and two oxygen donor atoms, which form a stable [Cu(II)(Gln)_2_] complex with a distorted (4 + 2) octahedral geometry [67]. Moreover, further EPR studies of a synthesised Cu(II)-glutamine complex demonstrated that the gradual exchange of glutamine-Cu(II) binding ligands amongst free and metal ion-bound forms in ^1^H NMR spectra arises from a weak exchange coupling (average exchange coupling constant = 0.42 K) [68]. Hence, this supports the observations made herein.

Indeed, glutamine has been recognised as a critical factor for the proliferation of various cell types (e.g., lymphocytes, hybridoma and neoplastic cells) in in vitro culture studies by functioning as a supplementary energy substrate, with special regard to the novel findings by Eagle et al. [69], which demonstrated that this particular amino acid is imperative for the proliferation of cell lineages characterised by fast regeneration, such as fibroblasts and HeLa cells. Thus, a glutamine deficiency may be perceived as a state of ‘nutritional stress’, applicable to both in vitro culture environments and in vivo settings.

The complexation of Cu(II) by glutamine has been demonstrated to be pivotal in boosting cellular interactions and possesses therapeutic efficacy. For example, in 2022, Yousuf et al. [34] reported that Cu(II)-glutamine complexes displayed a substantial anti-cancer efficacy, since research indicates that they possess the capacity to stimulate DNA damage and elevate intracellular ROS levels, ultimately resulting in the induction of apoptosis within tumour cells.

However, it is of much importance to note that glutamine is unstable in culture medium and decomposes to form pyroglutamate [26,27]. Indeed, the current study revealed that a significant proportion of the 2.055 mmol/L present, perhaps up to 50%, was degraded to pyroglutamate (Figure 2). This will undoubtedly affect the affinity of glutamine for metal ions, largely because pyroglutamate only has a limited complexing power when compared to that of its precursor. This arises because the cyclisation of its α-amino function into a lactam ring removes it as a key ligand donor site for metal ions. The remaining carboxylate group still has complexing capacity, but clearly this power is much weaker than that of the bidentate complexant glutamine itself.

Of particular relevance to the current study, and as noted above, recently Rewak-Soroczynska et al. [30] found that culture medium agents strongly affect the anti-bacterial actions of added copper, silver and zinc ions against *Pseudomonas aerugmasa*. To test this further, they investigated precipitates, solution turbidities and deposits formed between culture medium constituents and each of these metal ions. Notably, for both complex (Luria–Bertani Broth, Mueller Hinton Broth, Trypticase Soy Broth and Brain Heart Infusion Broth) and minimal media (MOPS, M9, M63 and Davis media), added Cu(II) formed such deposits, which were pale blue in colouration for this metal ion. Further work discovered that Davis medium and salts, HPO_4_^2−^, M9 medium salts, and M63 medium all gave rise to a major level of precipitation, whereas MOPS medium salts, tricine and H_2_PO_4_^−^ yielded a mild blue precipitate. Hence, for the current study, it appears that phosphate anions may be at least partially responsible for the pale blue-coloured deposit formed.

However, in Ref. [30], these researchers noted that in complex media only, an overnight agitation generated brown spots in culture wells from added Cu(II), a process accompanied by increases in turbidity. This phenomenon was largely explicable by the well- known redox reaction of Cu(II) with glucose, to form insoluble brown/red copper(I) oxide (Cu^I^_2_O) and gluconate in the culture medium (as with Fehling’s or Benedict’s solutions for the testing of urinary glucose); this reaction may occur at acidic and neutral, as well as alkaline pH values [70].

Hence, the adventitious removal of bactericidal Cu(II) through precipitation in this manner is largely responsible for the substantial decreases in minimum inhibitory concentrations (MICs) observed. In the current study, we were able to observe significant Cu(II)-dependent reductions in the intensities of the culture medium glucose resonances by up to 30%, but since the highest level of this metal ion added was only 67 µmol/L, and the total culture medium glucose concentration was 11.1 mmol/L (Table S1), its chemical consumption via this reaction will not account for it, and therefore added Cu(II)-mediated broadening of this signal is the most likely explanation for these results. Moreover, further culture medium reductants such as reduced glutathione (GSH) are unlikely to represent the source of insoluble Cu^I^_2_O, since its added level is only 3.26 µmol/L therein, and products from the reaction between this thiol and Cu(II) are likely to comprise a polymeric 1:1 Cu(I):thiolate complex with repeating bridging thiolate sulphur donors, as indeed it is for L-cysteine [71]. All of the cysteine residues present in the culture medium are present as the disulphide L-cystine anyway.

A further report [72] states that there were marked decreases in ‘free’ copper level on addition to culture media, and this clearly has striking implications for the current study in that added Cu(II) levels of ˂7 µmol/L may indeed be sufficient to broaden selected biomolecule resonances such as those of L-histidine.

Interestingly, the toxicity levels of Ni(II) and Cu(II) ions towards various cell lines have been previously investigated. For example, Taira et al. (2008) [73] performed an investigation to assess the ability of Ni(II) ions to exert cytotoxic effects towards a mouse embryonic fibroblast cell line C3H-10T1/2 utilising the Dulbecco’s Modified Eagle Medium culture medium; their findings exhibited a drop in the cell count number at Ni(II) concentrations up to 40 µmol/L over a period of six days in this medium, followed by their complete eradication at elevated levels of 2.0 mmol/L in a dosage-dependent manner.

More recently, Zhang et al. [5] assessed the cytotoxicity of Ni(II) ions on L929 mouse fibroblast cells grown in complete RPMI-1640 culture medium (at 37 °C, with 5% CO_2_ and 100% humidity) using an in vitro methyl thiazolyl tetrazolium (MTT) assay (a colorimetric assay system employed to evaluate cellular metabolic activity). Results acquired showed that both Ni(II) concentrations being tested in the study (100 and 200 μmol/L) exerted an impact on the cell viability. With increasing Ni(II) concentration levels, reductions in the cell multiplication rate of each of the Ni(II)-treated samples were observed, as well as extensive alterations in gene and protein expression arising from the toxic effects of this metal ion. Hence, this study verified the cytotoxic actions of Ni(II).

Moreover, in 2021, Witt et al. [74] studied the effect of surplus copper ion quantities in a human astrocytic cell line (CCF-STTG1) cultured using the RPMI-1640 medium supplemented with glutamine, foetal calf serum, and also some antibiotics (at 37 °C, 5% CO_2_ and 100% humidity). This investigation showed quite considerable cytotoxic effects of elevated levels of this metal ion on the CCF-STTG1 cell line after 48 h, eventually leading to mitochondrial dysfunction followed by ROS production. Since the cytotoxic actions exerted by both Ni(II) and Cu(II) ions will be expected to be critically dependent on their complexation (speciation) status, the investigations conducted in the current study are therefore clearly of much significance.

## 4. Materials and Methods

### 4.1. Materials and Sample Preparation for ^1^H NMR Analysis

Gibco™ RPMI 1640 Culture Medium, (modified with L-glutamine, HEPES buffer and phenol red) was obtained from ThermoFisher Scientific, Loughborough, UK Ltd. and stored at room temperature (20–22 °C). Analytical grade Copper(II)-sulphate pentahydrate (CuSO_4_.5H_2_O) and Nickel(II)-chloride hexahydrate (NiCl_2_.6H_2_O) and EDTA (disodium salt dihydrate) were purchased from Sigma-Aldrich Chemical Co., Ltd. (Gillingham, Dorset, UK), and stored at ambient temperature (20–22 °C). Aliquots of stock Cu(II) and Ni(II) solutions in HPLC-grade water were prepared at 0.10, 1.00 and 10.00 mmol/L concentrations, respectively. In the first set of ^1^H NMR spectral titration experiments, Ni(II) was added at final concentrations of 71, 140, 280, 410, 540 and 670 µmol/L, whereas in the next series of ^1^H NMR spectral titration experiments, Cu(II) was added at final concentrations of (1) 0.71, 1.4, 2.8, 4.1, 5.4 and 6.70 µmol/L; (2) 7.1, 14.0, 28.0, 41.0, 54.0 and 67.0 µmol/L; and (3) 71, 140, 280, 410, 540 and 670 µmol/L.

For each of the two ^1^H NMR spectral titration experiments, 7 × 1.50 mL volume Eppendorf tubes (Fisher Scientific Ltd., Loughborough, UK) containing a 1.00 mL volume of RPMI 1640 Culture Medium were set up and centrifuged for 10 min at 13,000 rpm (4 °C). During the centrifugation period, 7× fresh 1.50 mL Eppendorf tubes per titration experiment were labelled according to the study protocol involving 6 x tubes for each of the above-specified concentrations of Ni(II)_(aq_._)_ or Cu(II)_(aq_._)_, with the seventh tube serving as that for the untreated essential control sample, consisting only of RPMI 1640 culture medium supernatant and no added metal ions. Following centrifugation, 0.70 mL aliquots of RPMI 1640 culture medium supernatant samples were loaded into each of the six appropriately labelled tubes containing increasing volumes of stock Ni(II) or Cu(II) solutions, i.e., 5–50 µL via micropipette, which gave rise to the above specified final concentrations. Reaction mixtures were then thoroughly vortexed and centrifuged for 20 min at 13,000 rpm (4 °C).

Treatment of the culture medium with Cu(II), but not with Ni(II), was found to yield a pale blue-coloured solid deposit, which may comprise a Cu(II)-phosphate ([Cu^II^-PO_4_]^−^) and/or -carbonate ([Cu^II^-CO_3_]) material(s) (the culture medium contained 23.81 mmol/L sodium carbonate, although phosphate was present at a level of 5.63 mmol/L. Following thorough vortex mixing and centrifugation (10,000 r.p.m. at 4 °C), sufficient volumes of the clear supernatant were removed from the precipitate for speciation experiments using ^1^H NMR analysis. To enable these particulate materials to undergo ATR-FTIR analysis, they were thoroughly washed three-fold with ice-cold HPLC-grade water. Following washing, they were left to air dry in a dark laboratory environment at ambient temperature (>16 h overnight).

To selected replicate (n = 3) culture medium samples pre-treated with only 6.0 or 60 µmol/L Cu(II) or Ni(II) ions were subsequently added a final concentration of 60 µmol/L EDTA, and both sets of these were then equilibrated for periods of 0.50 or 24.0 h at ambient temperature prior to acquisition of their ^1^H NMR profiles.

Using glass pipettes (Fisher Scientific Ltd., Loughborough, UK), typically, 640 µL volumes of each control (untreated) or Ni(II) or Cu(II) ion-treated RPMI 1640 medium samples (at the above specified final concentrations) were transferred to 5 mm diameter NMR tubes, and then 60 µL of a 5.8 × 10^−3^ mol/L solution of 3-(trimethylsilyl)propionate-2,2,3,3-d_4_ (TSP, Sigma-Aldrich Chemical Co., Ltd., Gillingham, UK) in D_2_O, of purity 98 atom% deuterium (TSP), acting as a chemical shift reference (δ = 0.00 ppm) and internal quantitative ^1^H NMR standard, was added, and the sample mixtures were again vortex mixed. D_2_O served as a field frequency lock for ^1^H NMR experiments. Samples treated with EDTA following metal ion addition were also similarly prepared.

### 4.2. ^1^H NMR Measurements

Proton (^1^H) NMR measurements on the above prepared samples were recorded on a JEOL ECZ-600 MHz spectrometer (Leicester School of Pharmacy facility, De Montfort University, Leicester, UK) operating at a frequency of 600.17 MHz. Spectra were obtained in an automated mode, with suppression of the extremely intense H_2_O/HOD resonance (δ = 4.65–5.16 ppm), along with any rapidly relaxing signals arising from any culture medium macromolecules (although such species, e.g., proteins, were certainly not specified to be present), through utilisation of the WASTED pulse sequence. ^1^H NMR acquisition parameters were: 4 pre-scans, 256 scans, a sweep width of 12 ppm, a 1 s relaxation delay, 16,384 datapoints, and a probe operating temperature of 298.15 K. Chemical shifts were referenced to internal TSP, and all spectra were manually phased and baseline-corrected prior to further analysis. The -CH_3_ group resonances of acetate (*s*, δ = 1.92 ppm) and threonine (*d*, δ = 1.337 ppm) served as secondary internal chemical shift references in all non-Ni(II) and -Cu(II) samples investigated.

### 4.3. Analysis of ^1^H NMR Spectra

TSP normalisation, and where required, quantification of all ^1^H NMR-detectable compounds, was piloted based on the principle that the area of the signal resonances visible in the ^1^H NMR spectrum is directly proportional to the number of ^1^H nuclei giving rise to them [75]. Hence, for normalisation purposes, these intensities were divided by that of the TSP-Si(CH_3_)_3_ signal, and then multiplied by its known added concentration, and also by the factor 9/n, where n is the number of ^1^H nuclei of the analyte resonance. However, this application was limited in view of the paramagnetic metal ion-induced broadening of the TSP-Si(CH_3_)_3_ resonance [13], which occurred at the higher added levels, particularly for Cu(II).

The assignments pertaining to the majority of these resonances were established by detailed considerations of chemical shift values (δ/ppm) and coupling patterns (multiplicities), in conjunction with comparisons of their resonance profiles to those of verified literature and readily available database information, including that in the *Human Metabolome Database* 5.0 [28].

### 4.4. Preprocessing of ^1^H NMR Spectral Profiles

The identities of all culture medium biomolecule signals present in the ^1^H NMR profiles acquired were routinely assigned via a consideration of chemical shift values, coupling patterns, and coupling constants. Each of these was then cross-checked with actual or predicted 600 MHz spectra available in the HMDB. The RPMI 1640 culture medium sample dataset matrix (Table S1, Supplementary Information) was employed to facilitate resonance assignments. ^1^H NMR profiles were generated via the employment of macro procedures (JEOL Delta 5 software v 5.3.1). An exponential default line-broadening function of 0.20 Hz was applied to FIDs before their Fourier transformation to spectral profiles using zero-filling and autophase. Moreover, gradient shimming was applied using optimised shim values (standard JEOL NMR data format). The JEOL automatic polynomial baseline correction was applied to all spectra acquired.

Subsequently, an independent macro for a pre-fixed 0.04 ppm bucketing processing subroutine, was performed using the ACD/*Labs Spectrus Processor* 2022 and 2023 software packages (ACD/Labs, Toronto, ON, Canada). Before applying this fixed bucketing process, all spectral profiles were visually examined for any distortions, and were manually corrected, where required. The residual H_2_O/HOD signal (δ = 4.65–5.16 ppm) region remaining after application of the WASTED pulse sequence was removed from all spectral profiles prior to the performance of univariate and multivariate (MV) statistical analysis evaluations.

Fixed 0.04 ppm chemical shift buckets were used in this investigation rather than the usual ‘intelligent bucketing’ processing subroutine, since this allowed the possible observation of added Ni(II)- and Cu(II)-induced signal broadenings that stretched to directly adjacent resonance buckets, in addition to their original major ones. Hence, this analysis featured the measurement of decreases in the intensities of their original, untreated control sample resonance buckets arising from the effects of the added metal ions concomitantly with any corresponding increases in those located at lower or higher frequencies of the central 0.04 ppm region. Similarly, any added Ni(II)- or Cu(II)-induced, slow ligand-exchange modifications in metabolite signal δ values were also considered to be more easily detectable when using this type of bucketing, with the presumption that the change observed features their up- or downfield shifts to adjacent or even more remote paramagnetically shifted frequencies.

Therefore, this preprocessing approach gave rise to a global table of fixed 0.04 ppm bucket intensities, which was then transferred to MS Excel for additional manipulation. For univariate ANOVA and MV data analysis, fixed-width salivary bucket intensities were constant sum normalised (CSN) rather than normalised to that of the fixed concentration TSP internal standard resonance. However, in view of some added metal ion concentration-dependent complications with CSN [13], TSP signal normalisation was also conducted for comparative purposes.

### 4.5. FTIR-ATR Analysis of Deposit Formed from the Addition of Aqueous Cu(II) to RPMI 1640 Culture Medium

A Bruker ALPHA II-Platinum FT-IR spectrometer (Bruker UK Ltd., Coventry, UK) equipped with a universal platinum diamond-ATR QuickSnap sampling module was employed to perform reliable and rapid FTIR analysis of the Cu(II)-containing precipitate samples formed. Before all analyses, the spectrometer sample holder window was cleansed with an alcohol spray. A background spectrum was then recorded, before the Cu(II) deposit matrix derived from the addition of Cu(II) to the RPMI 1640 culture medium samples was then placed onto the sample holder window. All spectra were acquired within the scan range of 400–4000 cm^−1^ with a resolution of 2 cm^−1^ and a measurement period of 20 s with atmospheric compensation. Each analysis was conducted in triplicate. The interferogram size was 30,402 points.

In addition to the acquisition of ATR-FTIR spectra on the solid material arising from the addition of Cu(II), the residual culture medium solution samples present after centrifugation were isolated and analysed, in addition to untreated, control culture medium solutions. For this purpose, 0.70 μL aliquots of samples were left on the detection window to dry completely for a minimum period of 20 min until they had generated a thin film. ATR-FTIR spectra were then acquired in triplicate as described above.

### 4.6. Statistical and Metabolomics Analysis of Experimental Datasets

Univariate and MV statistical analysis techniques were conducted using the *MetaboAnalyst* 6.0 software option (University of Alberta and National Research Council, National Institute for Nanotechnology (NINT), Edmonton, AB, Canada). Prior to analysis, datasets were constant sum-normalised (CSN), generalised logarithmically (glog_10_)-transformed, and Pareto-scaled. As noted above, CSN was primarily applied in case of any added Ni(II)- and Cu(II)-induced resonance broadenings to the TSP internal standard resonance. However, in view of additional complications caused by the application of CSN, especially those observed at higher added metal ion concentrations, we also applied direct TSP-normalisation for univariate and MV analysis purposes.

One-way analysis of variance (ANOVA) was principally utilised to determine the false discovery rate (FDR)-corrected significance of differences between the mean ^1^H NMR bucket intensity values observed at increasing concentrations of added Ni(II) (71–670 µmol/L) and Cu(II) ions (7.1–67 µmol/L); in view of the very clear differences noted in the aromatic region, this ANOVA strategy was only applied to the high- and medium-field buckets, i.e., 0.90–5.40 ppm since added metal ion-induced modifications to those in the aromatic region were very clearly visible. FDR-corrected *p* values (Fisher’s least significant difference) were computed, and then violin plots were produced for all those which were statistically significant. Subsequently, a MV statistical analysis *MetaboAnalyst* 6.0 module was applied to perform significance analysis of metabolomics variables (^1^H NMR profile buckets) (SAM), and random forest (RF) analysis for both the Ni(II) and Cu(II) treatment groups. SAM analysis was conducted using a delta values of 22.7 and 1.7, and FDR values of 0.000 and 0.002 for Ni(II) and Cu(II) analyses, respectively, and cutup and cutdown parameters of 2.082 and -infinity. MV RF analysis was conducted with 500 trees, seven predictors per branch node, and an out-of-the-bag (OOB) error of 0.00.

### 4.7. Ethical Considerations

This study did not require ethics approval since it did not involve human participants, animal subjects, nor any sensitive data derived therefrom. Therefore, no ethical approvals were required in order to conduct this research programme.

## 5. Conclusions

In conclusion, the study reported herein offers valuable insights regarding the fate of low-molecular-mass Ni(II) and Cu(II) complexes within the RPMI 1640 mammalian cell culture medium, a study of much importance to those exploring the toxicities of these metal ions in cell culture experiments. Under the experimental conditions implemented, we detected Ni(II) and Cu(II) coordination by amino acids (specifically, histidine, glutamine, acetate, methionine, lysine, and further amino acids interacting with both Ni(II) and Cu(II)), with histidine being the most powerful complexant available. Although a minimal concentration of 71 µmol/L Ni(II) was required to selectively enhance the linewidths of histidine’s imidazole ring protons, only 7.1 µmol/L of added Cu(II) was needed for a complete broadening of these resonances within the culture medium, and this confirms that histidine, along with further transition metal ion-complexing amino acids, are ca. 10-fold more sensitive to the paramagnetism of Cu(II) than they are to that of added Ni(II) ions, the latter being coordination geometry- and ligand field strength-dependent. However, these results confirm the preferential complexation of both these metal ions by this amino acid and also highlight its potential critical role in Ni(II) and Cu(II) homeostasis in vivo. Additionally, in the high-field region of spectra acquired, the addition of low concentrations of Cu(II) also resulted in the disappearance of the glutamine and BCAA resonances, and organic acid anion signals (e.g., that of acetate), together with those of further amino acids (e.g., lysine and aspartate). The predominant nature of complexation for Cu(II) and Ni(II) ions in this medium may affect and/or modulate their toxicological effects when present at excessive levels. Treatment of the culture medium with Cu(II) gave rise to a deposit, which may comprise a solid Cu(II)-phosphate or -carbonate species (Cu(II)-oxide and -hydroxide species were ruled out), although it is also likely to contain at least some Cu(I)_2_O arising from redox reactions involving glucose. Hence, it is crucial to conduct further research into the specific status of these physiologically formed complexes, (e.g., by employing further techniques including spectrophotometry, ATR-FTIR and X-ray crystallography), and their associated chemistries regarding Ni(II)/Cu(II)-complexation capabilities and ROS-generating or -scavenging activities (e.g., through molecular ‘docking’ studies, density functional theory and EPR spectroscopy), in addition to investigating their thermodynamic coordination stability via thermal and computational simulation analysis. Such investigations will serve to further our understanding of these phenomena.

## Figures and Tables

**Table 1:** Assignments of resonances in the 600.17 MHz ^1^H NMR spectra of supernatants of RPMI 1640 culture medium samples before treatment with increasing concentrations of nickel(II) and copper(II) ions (chemical shift (δ/ppm) values and resonance coupling patterns are also shown).

Signal Code	Chemical Shift (δ/ppm)	Coupling Pattern (Multiplicity)	Corresponding Biomolecule (Assignment)	ANOVA Significance (*p*) Level with Ni(II) Treatment (0.90–5.40 ppm Only)	ANOVA Significance (*p*) Level with Cu(II) Treatment (0.90–5.40 ppm Only)
**1**	0.965	*d*	Leucine-CH_3_	>6.10 × 10^−13^ ↑	>4.44 × 10^−3^ ↓
**2**	0.990	*d*/*t*/*d*	Leucine-CH_3_/Isoleucine-γ-CH_3_/Valine-CH_3_	4.14 × 10^−16^	1.38 × 10^−42^ ↓
**3**	1.022	*d*	Isoleucine-β-CH_3_	8.26 × 10^−15^	3.04 × 10^−47^ ↓
**4**	1.041	*d*	Valine-CH_3_	6.10 × 10^−13^ ↓	8.02 × 10^−53^ ↓
**5**	1.337	*d*	Threonine-CH_3_	6.09 × 10^−20^ ↓	2.01 × 10^−24^
**6**	1.461	*m*	Lysine-γ-CH_2_	3.54 × 10^−18^ ↑	>4.44 × 10^−3^ ↓
**7**	1.644	*m*	Arginine-β-CH_2_	>6.10 × 10^−13^	>4.44 × 10^−3^ ↓
**8**	1.674	*m*	Leucine-CH_2_	1.43 × 10^−31^ ↓	7.94 × 10^−37^ ↓
**Lys**	1.720	*tt*	Lysine-δ-CH_2_	3.16 × 10^−21^ ↓	2.12 × 10^−37^ ↓
**8A**	1.896	*td*	Lysine-β-CH_2_	>6.10 × 10^−13^	>4.44 × 10^−3^
**9**	1.915	*s*	Acetate-CH_3_	2.57 × 10^−25^	2.39 × 10^−54^
**9A**	1.919	*m*	Arginine-γ-CH_2_	2.57 × 10^−25^ ↓	2.39 × 10^−54^ ↓
**10**	2.000–2.070 (2 or more partially resolved signals)	*m*/*s*	Pyroglutamate-1/2-β-CH_2_/Carbohydrate N-Acetylsugar-NHCOCH_3_ functions/Hydroxyproline-1/2-β-CH_2_	1.48 × 10^−25^ ↑	3.81 × 10^−17^ ↓
11 *	2.046	*sharp s*	Free N-Acetylated biomolecule–NHCOCH_3_	5.59 × 10^−18^ ↑	1.76 × 10^−16^ ↓
**11A**	2.074	*sharp s*	Free O-Acetylated biomolecule-OCOCH_3_	5.59 × 10^−18^ ↑	1.76 × 10^−16^ ↑
**12**	2.141	*s*	Methionine-SCH_3_	1.62 × 10^−43^ ↑	>4.44 × 10^−3^ ↓
**13**	2.353	*m*	Glutamate-γ-CH_2_/Proline-β-CH_2_/Pyroglutamate-γ-CH_2_	2.36 × 10^−27^	>4.44 × 10^−3^
**14A**	2.396	*m*	Unassigned part-added Ni(II)- and Cu(II)-resistant multiplet, possibly superimposed on weak Pyruvate-CH_3_ (*s*) and/or Succinate-CH_2_ (*s*) resonances/Pyroglutamate-γ-CH_2_	1.48 × 10^−28^	>4.44 × 10^−3^ ↓
**14B**	2.433	*m*	Glutamine-γ-CH_2_	1.48 × 10^−28^ ↑	2.75 × 10^−42^ ↑
**14C**	2.503	*m*	Pyroglutamate-1/2-β-CH_2_/Hydroxyproline-1/2-β-CH_2_	3.38 × 10^−19^ ↑	2.75 × 10^−42^ ↓
**n/a**	2.602	*m*	Methionine-γ-CH_2_	3.38 × 10^−19^	4.44 × 10^−3^ ↑
**n/a**	2.682	*dd*	Aspartate ½ β-CH_2_	6.98 × 10^−34^	>4.44 × 10^−3^
**n/a**	2.84	*m*	Asparagine ½ β-CH_2_	2.47 × 10^−45^ ↓	4.71 × 10^−16^ ↓
**n/a**	2.94	*m*	Asparagine ½ β-CH_2_	2.09 × 10^−45^ ↓	3.65 × 10^−32^ ↓
**15**	3.035	*t*	Lysine-ε-CH_2_	7.33 × 10^−22^ ↓	5.74 × 10^−23^
**16**	3.106	*s*	Dimethylsulphone SO(CH_3_)_2_	9.61 × 10^−14^	2.99 × 10^−21^
**17**	3.205	*s*	Choline-N(CH_3_)_3_^+^	>6.10 × 10^−13^	>4.44 × 10^−3^ ↑
**18**	3.263	*t*	β-Glucose-C2H	3.21 × 10^−25^	6.72 × 10^−21^
**19**	3.392	*s*	Methanol-CH_3_	1.09 × 10^−24^ ↓	3.29 × 10^−14^
**20**	3.419	*dd*	α-Glucose-C4H/Proline-α-C1H	1.78 × 10^−20^ ↓	2.68 × 10^−35^
**21**	3.469	*dt*	β-Glucose-C5H	>6.10 × 10^−13^ ↑	1.15 × 10^−15^
**22**	3.495	*dd*	β-Glucose-C3H	9.97 × 10^−17^ ↑	2.14 × 10^−17^
**23**	3.536	*dd*	α-Glucose-C2H	6.84 × 10^−18^	3.65 × 10^−16^
**24**	3.564	*s*	Glycine-CH_2_	1.49 × 10^−13^	5.86 × 10^−16^
**25**	3.600	*m*	Valine-α-CH	1.17 × 10^−17^ ↓	6.21 × 10^−18^
**26**	3.704	*s/m/t*	Unknown/Leucine-α-CH/Lysine-α-CH	7.68 × 10^−34^ ↑	4.05 × 10^−9^
**27**	3.719	*dd*	α-Glucose-C3H^′^	7.68 × 10^−34^ ↑	2.11 × 10^−22^ ↑
**28**	3.737	*dd*	β-Glucose-C6H_b_	3.66 × 10^−19^	8.88 × 10^−15^
**29**	3.778	*dt*	α-Glucose-C6H^′^_b_	>6.10 × 10^−13^	>4.44 × 10^−3^
**30**	3.898	*dd*	β-Glucose-C6H_a_	>6.10 × 10^−13^ ↓	>4.44 × 10^−3^ ↓
**n/a**	4.005	*dd*	Asparagine-α-CH	2.11 × 10^−26^ ↓	9.60 × 10^−39^ ↓
**31**	4.081	*2 × t*	Cystine-α-CH	1.37 × 10^−16^ ↓	5.94 × 10^−10^ ↓
**32**	4.188	*q*	Pyroglutamate-α-CH	5.98 × 10^−36^	1.41 × 10^−15^
**33**	5.237	*d*	α-Glucose-C1H	1.12 × 10^−16^	5.74 × 10^−23^ ↑
4-HPA *	6.685	*d*	* 4-Hydroxyphenylacetate-CH_2_	na	na
**34**	6.894	*d*	Tyrosine aromatic ring-C2H/C6H	na	na
**35**	7.058	*s*	Histidine imidazole ring-C4H	na	na
**36**	7.190	*d*	Tyrosine aromatic ring-C3H/C5H	na	na
**37**	7.328	*m*	Phenylalanine aromatic Ring-C2H/C6H	na	na
**38**	7.382	*m*	Phenylalanine aromatic ring-C4H	na	na
**39**	7.435	*m*	Phenylalanine aromatic ring-C3H/C5H	na	na
**39A**	7.548	*d*	Tryptophan aromatic ring-C2H	na	na
**39B**	7.743	*d*	Tryptophan aromatic ring-C3H	na	na
**40**	7.770	*s*	Histidine imidazole ring-C2H	na	na

* Indicates a tentative assignment. Assignments pertaining to the majority of these resonances were established by detailed considerations of chemical shift values (δ/ppm) and coupling profiles (multiplicities), in conjunction with the comparisons to verified literature and readily available database information, including the *Human Metabolome Database* [28]. All chemical shift values were referenced to TSP (δ = 0.00 ppm). Abbreviations: *s*, *d*, *dd*, *t* and *m* denote singlet, doublet, double doublet, triplet, and multiplet resonance multiplicities, respectively; na, not applicable. Assignments for α- and β-Glucose were verified by checking with those provided in Roslund et al. (2008) [29]. One-way ANOVA *p* values obtained for the 0.90–5.40 ppm region only are also provided for experiments involving spectral titrations of the culture medium with Ni(II) (71–670 µmol/L) and Cu(II) (7.1–67 µmol/L). The arrows ↓ and ↑ refer to cases where clear decreases or increases in the intensities of these resonances, respectively, were observable.

**Table 2:** Random Forest classification of culture medium samples titrated with increasing concentrations of Ni(II) and Cu(II) (the latter in brackets).

	Added [Ni(II] ([Cu(II)]) (µmol/L)							
**Added [Ni(II)] ([Cu(II)]) (µmol/L)**	**0.0 (0.0)**	**71 (7.1)**	**140 (14)**	**280 (28)**	**410 (41)**	**540 (54)**	**670 (67)**	**Class Error**
	**71 (7.1)**	4 (4)	0 (0)	0 (0)	0 (0)	0 (0)	0 (0)	0.00 (0.00)
	**140 (14)**	0 (0)	4 (4)	0 (0)	0 (0)	0 (0)	0 (0)	0.00 (0.00)
	**280 (28)**	0 (0)	0 (0)	4 (4)	0 (0)	0 (0)	0 (0)	0.00 (0.00)
	**410 (41)**	0 (0)	0 (0)	0 (0)	4 (4)	0 (0)	0 (0)	0.00 (0.00)
	**540 (54)**	0 (0)	0 (0)	0 (0)	0 (0)	4 (4)	0 (0)	0.00 (0.00)
	**670 (67)**	0 (0)	0 (0)	0 (0)	0 (0)	0 (0)	4 (4)	0.00 (0.00)

## Data Availability

Data presented in this study are available through contact with the correspondence author of this article (email: mgrootveld@dmu.ac.uk).

## Supplementary Materials

The following supporting information can be downloaded at: https://www.mdpi.com/article/10.3390/molecules31010085/s1. Section S1: Molecular Composition of the RPMI 1640 Culture Medium; Section S2: Violin Plots of Bucketed ^1^H NMR Data, both Normalised and Unnormalized; Section S3. Influence of Added Copper(II) Ions on the Linewidth of the TSP Internal Standard Resonance (δ = 0.00 ppm), and the Statistical Significance of TSP-Normalised Culture Medium Biomolecule Signal Intensities Arising from the Addition of Increasing Concentrations of Ni(II) and Cu(II) Ions (via one-way ANOVA); Section S4: Further investigations of the ATR-FTIR Spectra of the Deposit Generated from the Interaction of Cu(II) with RPMI 1640 Culture Medium. References [76,77,78,79,80,81,82,83,84] are cited in the Supplementary Materials.
