# Supplementary material for: High-Resolution 1H NMR Investigation of the Speciation Status of Nickel(II) and Copper(II) Ions in a Cell Culture Medium: Relevance to Their Toxicological Actions
Source: Molecules. 2025 Dec 24;31(1):85. doi: 10.3390/molecules31010085 (PMC12786676; doi:10.3390/molecules31010085)
Supplement: Supplementary file 1 [file molecules-31-00085-s001.zip › molecules-3976700-supplementary.pdf]

High-Resolution ^{1}H NMR Investigation of the Speciation Status of Nickel(II) and Copper(II) Ions in a Cell Culture Medium: Relevance to their Toxicological Actions

Deepinder K. Kalra, Kayleigh Hunwin, Katie Hewitt, Olivia Steel and Martin Grootveld*

Leicester School of Pharmacy, De Montfort University, The Gateway, Leicester LE1 9BH, United Kingdom

SUPPLEMENTARY INFORMATION

***Correspondence to: Professor Martin Grootveld**

Email: mgrootveld@dmu.ac.uk

Section S1: Molecular Composition of the RPMI 1640 Culture Medium

Table S1. Molecular composition of the RPMI 1640 culture medium, with concentrations of amino acids, vitamins, metal ion salts and buffers, and further agents (glucose, glutathione and phenol red dye) provided in mmol/L units (adapted from Ref. [76])

**Agents**	**Concentration (mmol/L)**
**Amino Acids**	
Glycine	0.133
L-Arginine	1.149
L-Asparagine	0.379
L-Aspartate	0.150
L-Cystine.2HCl	0.208
L-Glutamate	0.136
L-Glutamine	2.055
L-Histidine	0.097
L-Hydroxyproline	0.153
L-Isoleucine	0.382
L-Leucine	0.382
L-Lysine hydrochloride	0.274
L-Methionine	0.101
L-Phenylalanine	0.091
L-Proline	0.174
L-Serine	0.286
L-Threonine	0.168
L-Tryptophan	0.025
L-Tyrosine (disodium salt dehydrate)	0.111
Valine	0.171
**Vitamins**	
Biotin	8.20×10^{-4}
Choline chloride	0.021
D-Calcium pantothenate	5.24×10^{-4}
Folic Acid	2.27×10^{-3}

Niacinamide	8.20 x 10^{-3}
Para-Aminobenzoic Acid	7.30 x 10^{-3}
Pyridoxine hydrochloride	4.85 x 10^{-3}
Riboflavin	5.32 x 10^{-4}
Thiamine hydrochloride	2.97 x 10^{-3}
Vitamin B12	3.69 x 10^{-6}
L-Inositol	0.194
**Metal Ion Salts and Buffers**	
Calcium nitrate $Ca(NO_3)_2.4H_2O$	0.424
Magnesium Sulphate ($MgSO_4$) (anhydrous)	0.407
Potassium Chloride (KCl)	5.333
Sodium Bicarbonate ($NaHCO_3$)	23.810
Sodium Chloride (NaCl)	91.379
Sodium Phosphate dibasic (Na_2HPO_4) (anhydrous)	5.634
HEPES Buffering Agent	25.034
**Further Agents (Carbohydrates, Trace Antioxidants, Dyes, etc.)**	
D-Glucose	11.111
Glutathione (reduced, GSH)	3.26 x 10^{-3}
Phenol Red	1.33 x 10^{-2}

Section S2: Violin Plots of Bucketed ^{1}H NMR Data, both Normalized and Unnormalized.

Violin plots of median ^{1}H NMR Signal Intensities for the 0.96-1.00, 1.00-1.04, 1.04-1.08, 1.48-1.52 (lysine), 1.68-1.72, 1.72-1.76, 1.92-1.96, 2.80-2.84 and 3.20-3.24 ppm buckets, both raw (left-hand-side) and 'normalised' (right-hand-side), *versus* added Cu(II) concentration (mmol/L) are shown below. The 'normalised' datasets on the right-hand-sides were constant sum-normalised (CSN), generalised logarithmically (glog)-transformed to the power 10, and then Pareto-scaled prior to analysis.

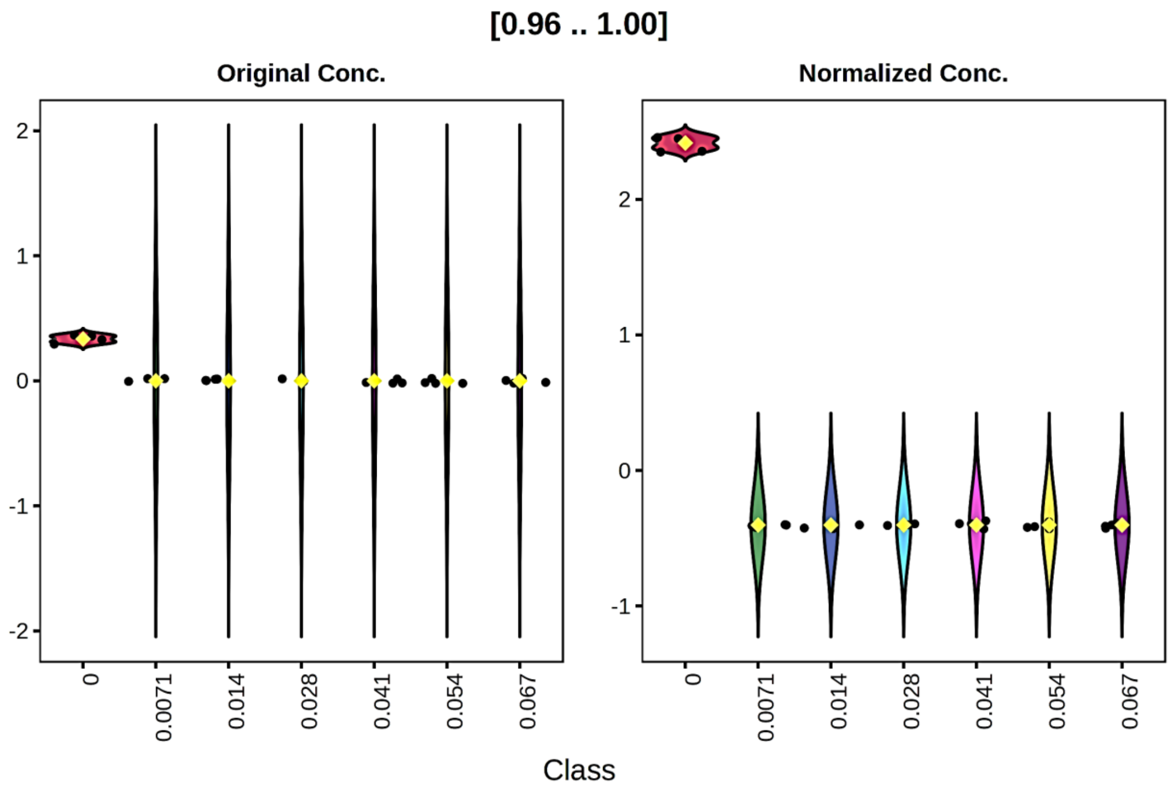

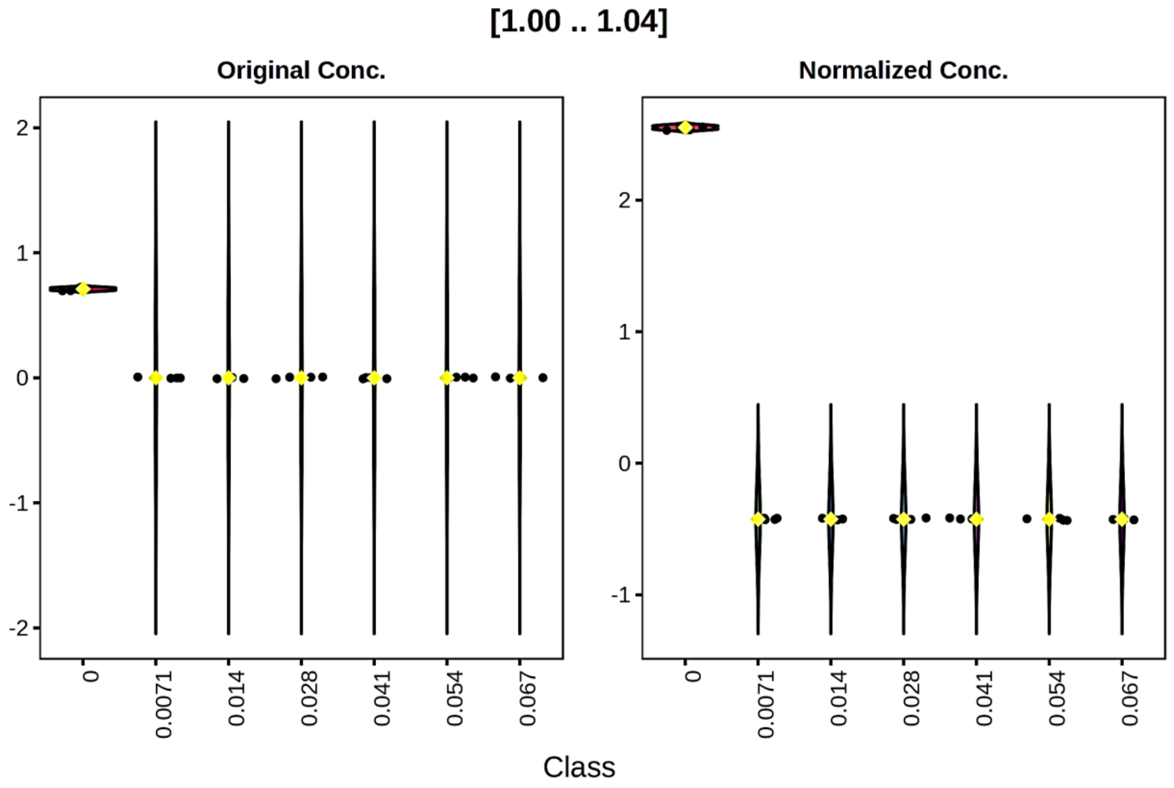
[1.00 .. 1.04]
Original Conc.
Normalized Conc.
2
1
0
-1
-2
0
0.0071
0.014
0.028
0.041
0.054
0.067
Class

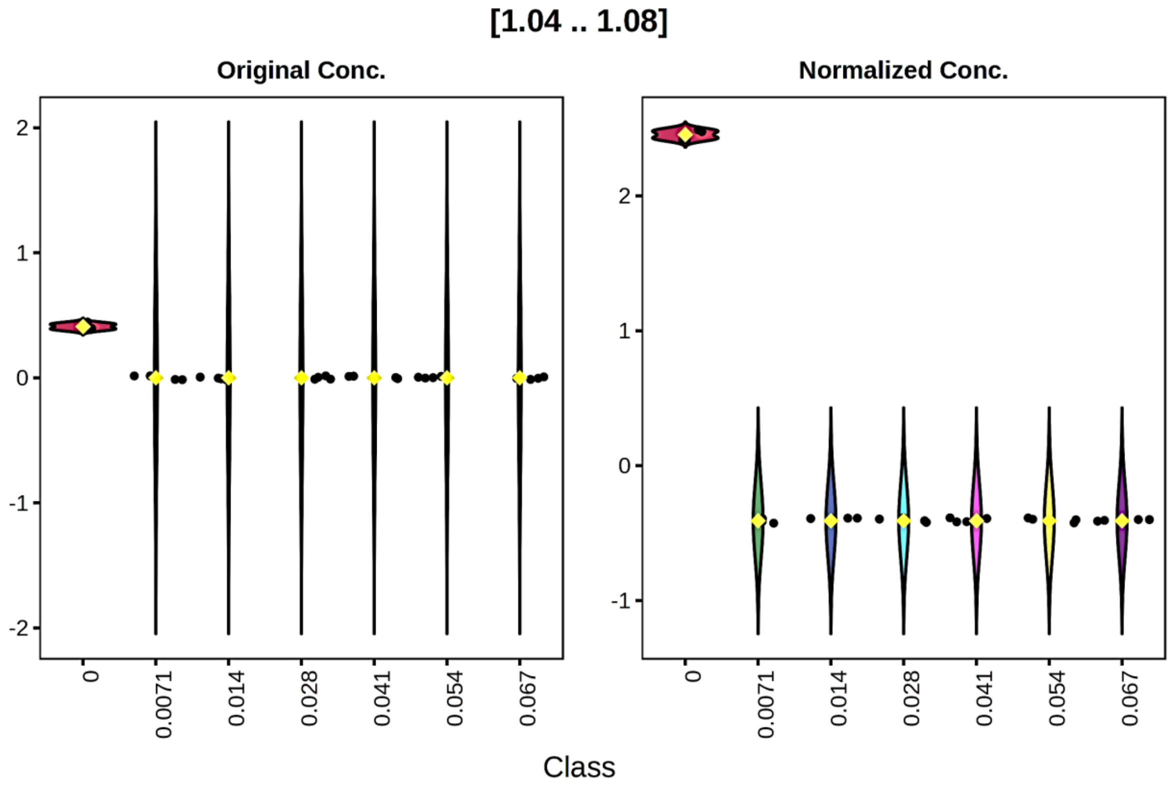
[1.04 .. 1.08]
Original Conc.
Normalized Conc.
2
1
0
-1
-2
0
0.0071
0.014
0.028
0.041
0.054
0.067
Class

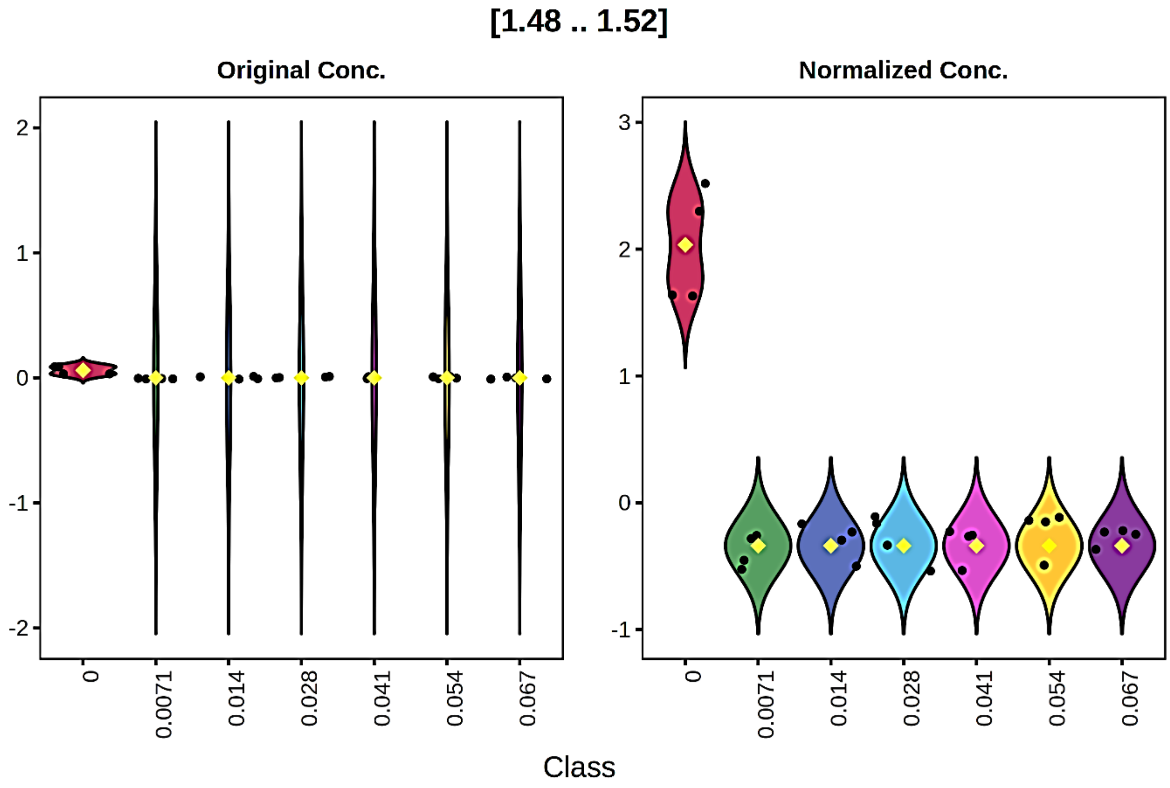
[1.48 .. 1.52]
Original Conc.
Normalized Conc.
2
1
0
-1
-2
3
0
0.0071
0.014
0.028
0.041
0.054
0.067
Class

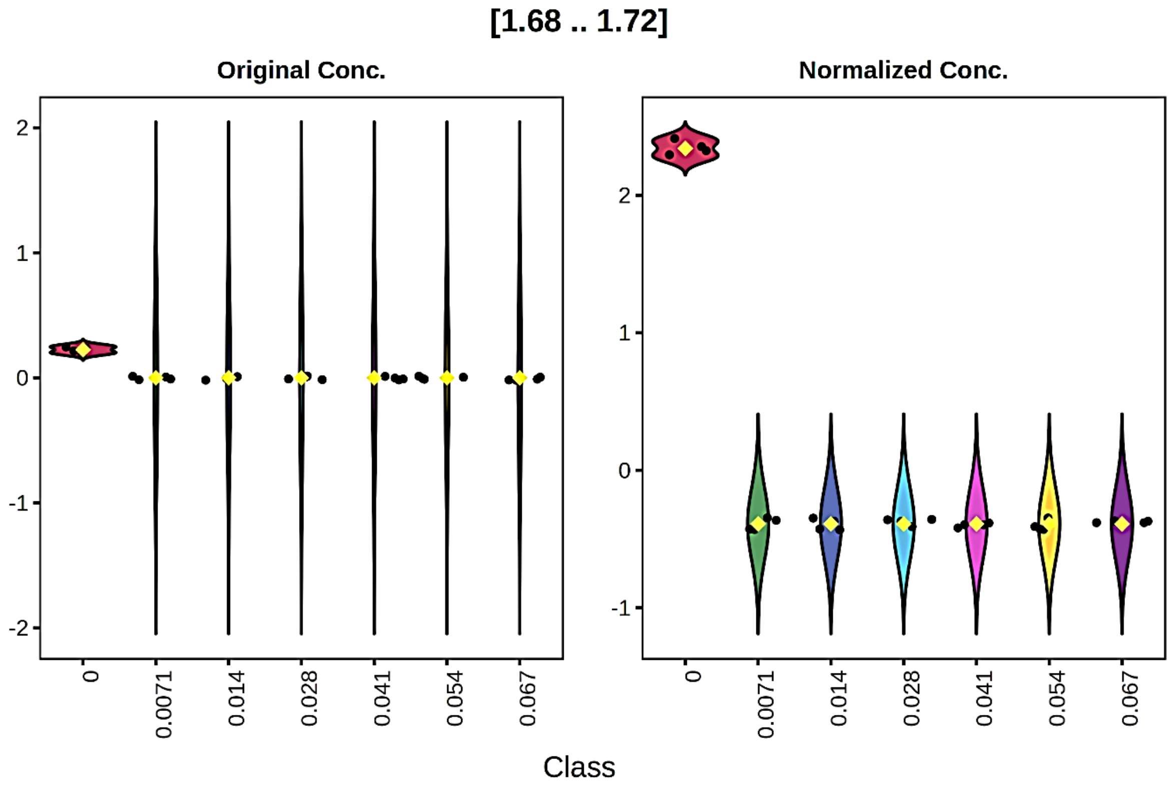
[1.68 .. 1.72]
Original Conc.
Normalized Conc.
2
1
0
-1
-2
0
0.0071
0.014
0.028
0.041
0.054
0.067
Class

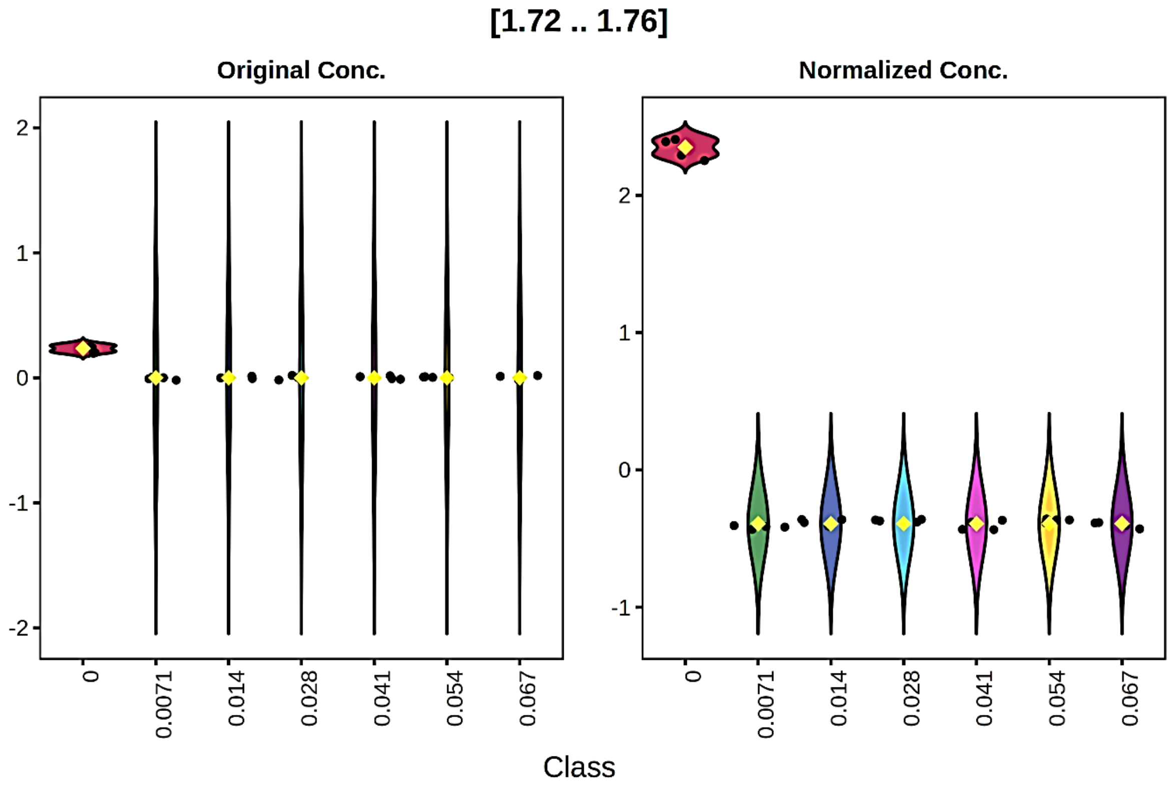
[1.72 .. 1.76]
Original Conc.
Normalized Conc.
2
1
0
-1
-2
0
0.0071
0.014
0.028
0.041
0.054
0.067
Class

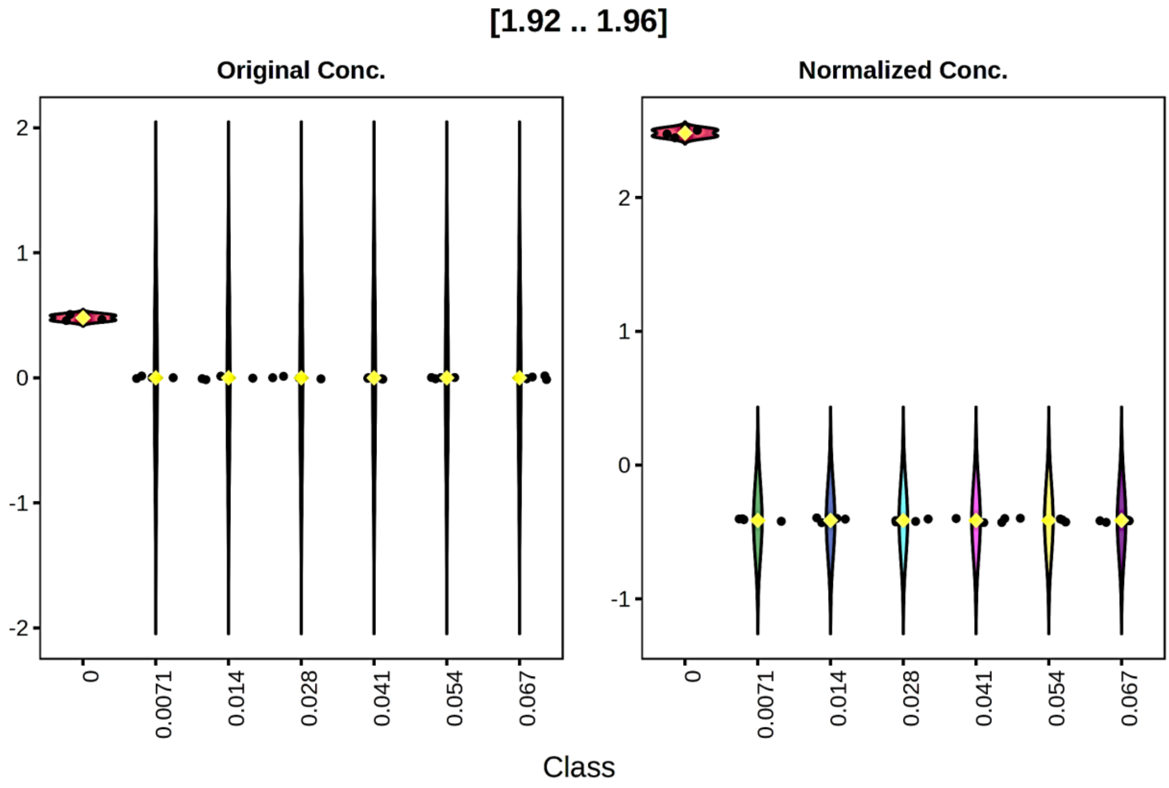
[1.92 .. 1.96]
Original Conc.
Normalized Conc.
2
1
0
-1
-2
0
0.0071
0.014
0.028
0.041
0.054
0.067
Class

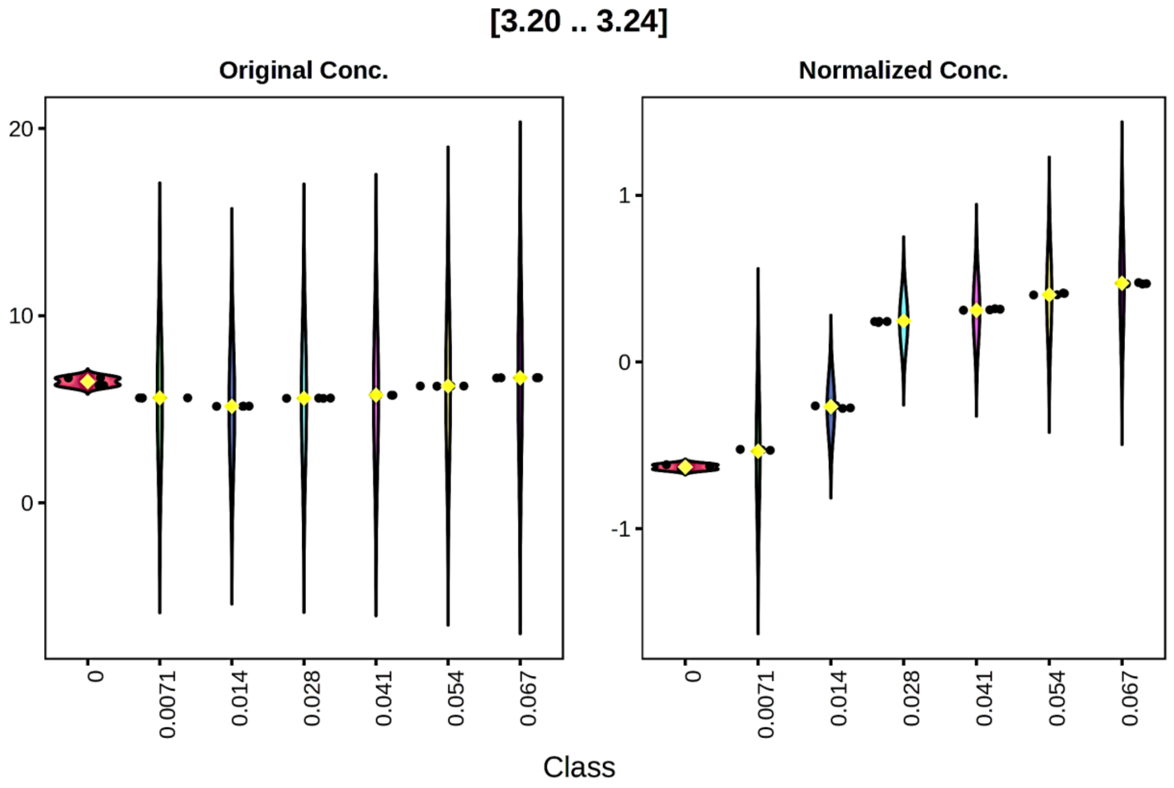

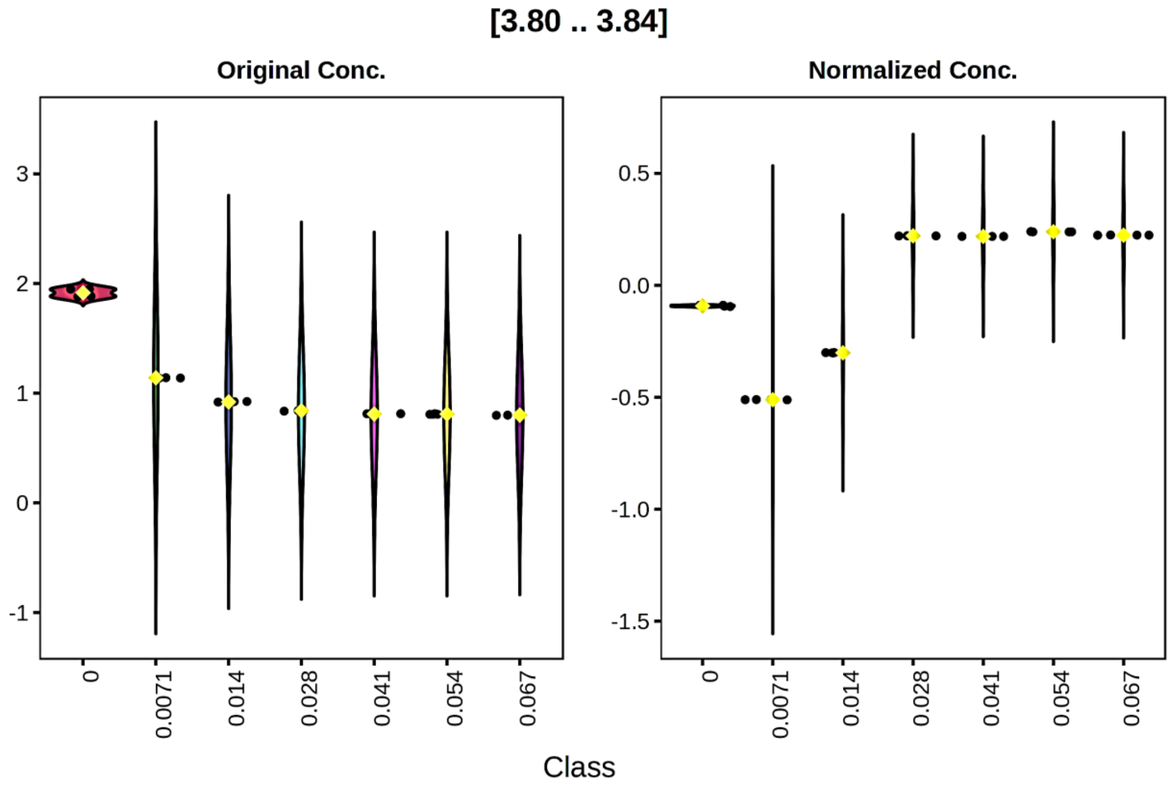

Figure S1. Violin plots of raw (left) and normalised bucket resonance intensities (each proportional to culture medium concentration, abbreviated Conc. (right)) *versus* added Cu(II) ion concentration for ^{1}H NMR spectral titrations of RPMI 1640 culture medium with this metal ion throughout the 0.0071 to 0.067 mmol/L range. Data shown is that for the 0.96-1.00 (Leucine-C<u>H</u>$_3$/Isoleucine-C<u>H</u>$_3$/Valine-C<u>H</u>$_3$), 1.00-1.04 (Isoleucine-C<u>H</u>$_3$), 1.04-1.08 (Valine-C<u>H</u>$_3$), 1.48-1.52 (Lysine-γ-C<u>H</u>$_2$), 1.68-1.72 (Leucine-

C$\underline{H}_2$), 1.72-1.76 (Lysine-β-CH_2), 1.92-1.96 (Acetate-CH_3), 2.80-2.84 (Asparagine, ½-β-CH_2), 3.20-3.24 (Choline-N(C$\underline{H}_3$)$_3^+$), and 3.80-3.84 ppm (α-Glucose-C6$\underline{H}'_b$) buckets. The 'normalisation' strategy involved constant sum normalisation (CSN), generalised logarithmic (glog)-transformation to the power 10, and Pareto scaling. The abscissa axis represents increasing added Cu(II) ions in mmol/L. ^{1}H NMR buckets depicted as [0.96 .. 1.00], for example, correspond to those which are 0.96-1.00 ppm, etc. Violin plots reveal the probability density of each dataset at distribution different values, which is smoothed by a kernel density estimator (KDE). The yellow diamond points represent markers for median values. The thick KDE line represents the boundary for the datapoint distribution. Usually, datapoints lying outside a quartile 3 (Q3) ± 1.5 inter-quartile range (IQR) limit are considered to be outliers.

Section S3. Influence of Added Copper(II) Ions on the Linewidth of the TSP Internal Standard Resonance (δ = 0.00 ppm), and the Statistical Significance of TSP-Normalized Culture Medium Biomolecule Signal Intensities Arising from the Addition of Increasing Concentrations of Ni(II) and Cu(II) Ions (via one-way ANOVA)

For experiments involving the TSP internal standard involved, we elected to explore plots of ratios of culture medium biomolecule resonance intensities to that of this ^{1}H NMR internal standard for both increasing Ni(II) and Cu(II) level treatments. However, prior to this, we monitored the line-width of the TSP resonance to evaluate its dependence on added Cu(II) concentration. Figure S2 shows the influence of increasing levels of added Cu(II) on the ^{1}H NMR TSP internal standard TSP-Si(C$\underline{H}_3$)$_3$ resonance intensity (final concentration 497 µmol/L), and from this it is clear that the line-width at half-height ($v_{1/2}$) value of this signal increases with increasing added concentrations of this metal ion. Moreover, Figure S3 shows a plot of $\Delta v_{1/2}$ versus added Cu(II) concentration for an experiment involving this assessment, and it was observed that there was a significant, approximately linear elevation in the dependent variable with increasing Cu(II) level (r = 0.9877, p <10^{-6}).

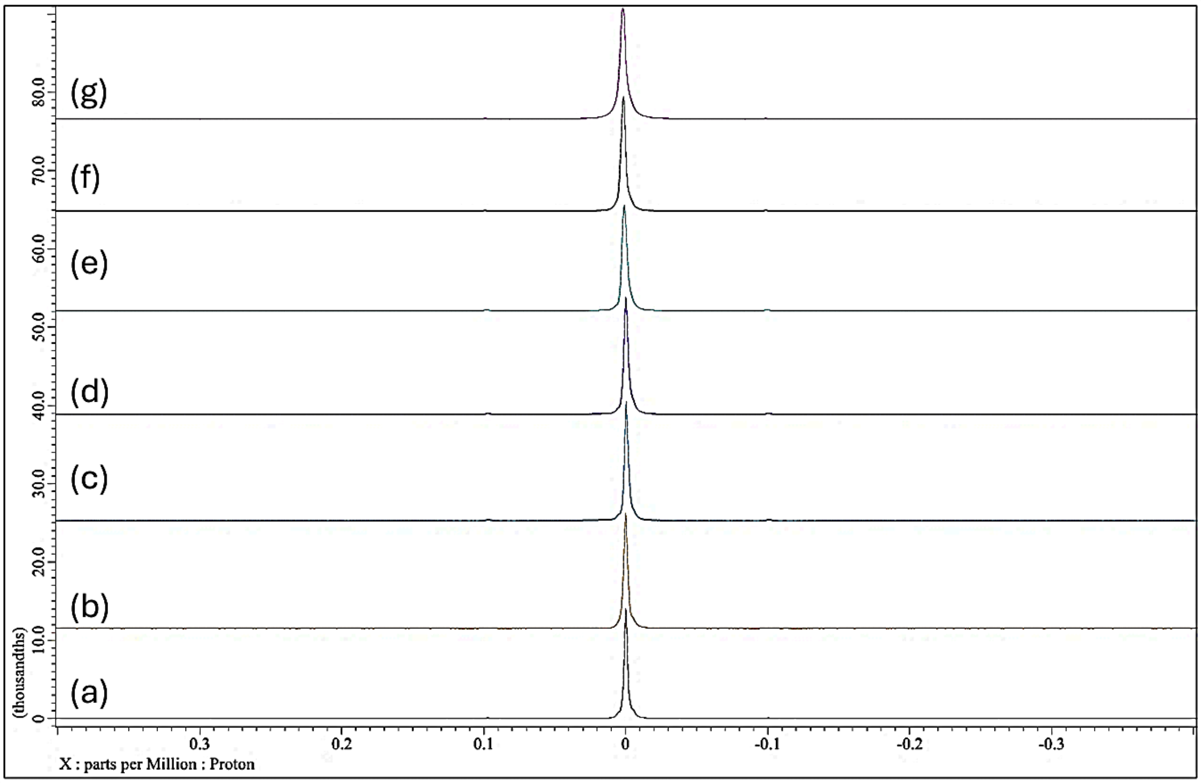

Figure S2. -0.40 to +0.40 region of the ^{1}H NMR spectra of RPMI 1640 culture medium treated with (a) 0.0, (b), 7.1, (c) 14, (d) 28, (e) 41, (f) 54 and 67 µmol/L Cu(II) These spectra show only the TSP -Si(C$\underline{H}_3$)$_3$ resonance, which steadily broadens from 1.58 Hz at no added Cu(II), to 3.21 Hz at an added level of 67 µmol/L. The TSP concentration was 497 µmol/L throughout

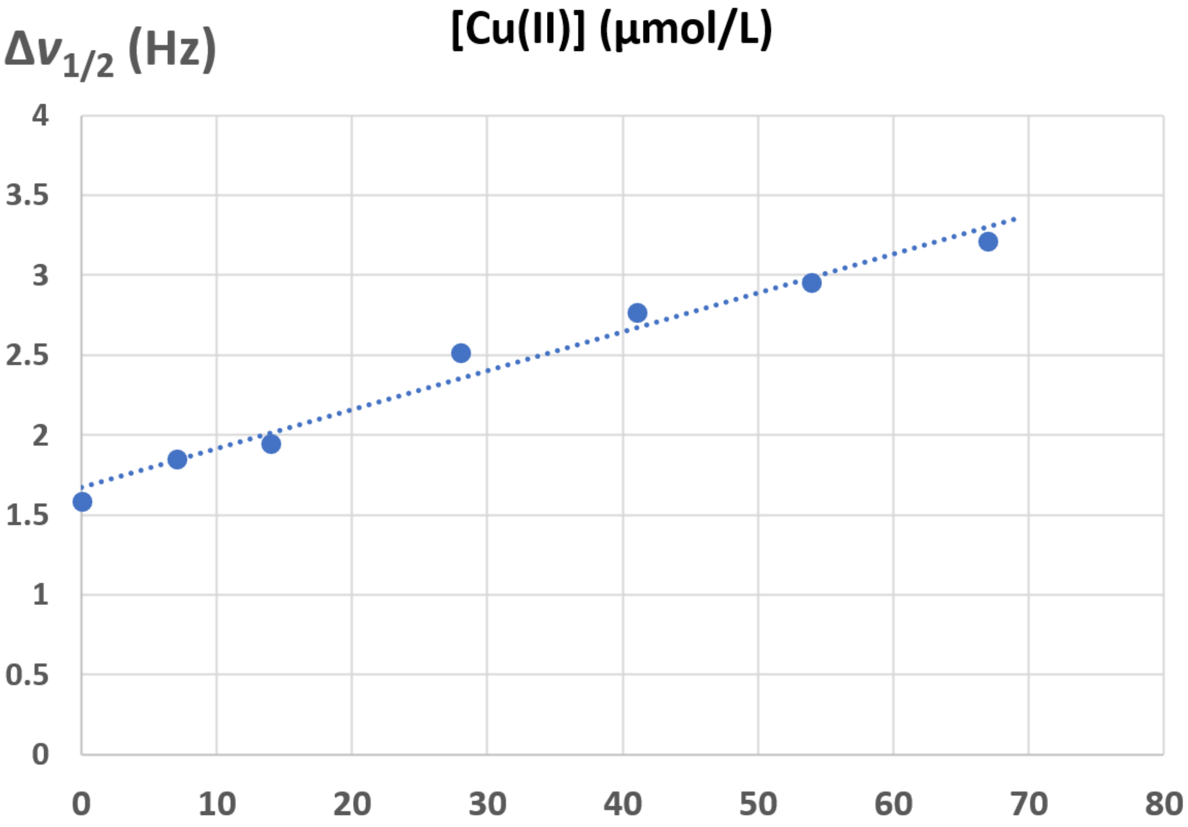

Figure S3. Plot of $\Delta v_{1/2}$ (Hz) versus Cu(II) concentration for the TSP-Si$(CH_3)_3$ resonance present in RPMI 1640 culture medium (data acquired from Figure S2), showing an approximate linear increase in line-width at half-height with increasing added level of this paramagnetic metal ion. The final analytical TSP concentration was 497 µmol/L.

Table S2. Ranked ANOVA-determined *p* values for the added Ni(II) ion concentration dependence of each TSP-normalised ^{1}H NMR bucket intensities within the 0.90-5.40 ppm chemical shift range (0-670 µmol/L added Ni(II)). The TSP internal standard concentration was 497 µmol/L. Resonance assignments are also provided. The false discovery rate (FDR) correction applied was Fisher's least significant difference test.

^{1}H NMR Bucket (ppm)	Assignment(s)	ANOVA FDR-Corrected *p* Value
[1.96 .. 2.00]	Arginine-γ-C<u>H</u>$_2$	3.1×10^{-191}
[1.52 .. 1.56]	Lysine-γ-C<u>H</u>$_2$	4.7×10^{-190}
[2.32 .. 2.36]	Glutamate-γ-C<u>H</u>$_2$/Proline-β-C<u>H</u>$_2$/Pyroglutamate-γ-C<u>H</u>$_2$	4.7×10^{-190}
[2.44 .. 2.48]	Glutamine-γ-C<u>H</u>$_2$	9.4×10^{-190}
[3.68 .. 3.72]	Unknown/Leucine-α-C<u>H</u>/Lysine-α-C<u>H</u>	1.2×10^{-189}
[1.68 .. 1.72]	Leucine-C<u>H</u>$_2$	4.6×10^{-189}
[3.28 .. 3.32]	β-Glucose-C2<u>H</u>	1.3×10^{-188}
[3.48 .. 3.52]	β-Glucose-C5H	1.3×10^{-188}

[3.60 .. 3.64]	Valine-α-CH	1.3×10^{-188}
[2.72 .. 2.76]	Aspartate ½ β-CH_2	2.1×10^{-188}
[1.92 .. 1.96]	Acetate-CH_3	2.1×10^{-188}
[2.52 .. 2.56]	Pyroglutamate-1/2-β-CH_2;/Hydroxyproline-1/2-β-CH_2	2.9×10^{-188}
[2.12 .. 2.16]	Methionine-SCH_3	6.2×10^{-188}
[2.68 .. 2.72]	Aspartate ½ β-CH_2	1.4×10^{-187}
[3.08 .. 3.12]	Dimethylsulphone $SO(CH_3)_2$	1.5×10^{-187}
[2.80 .. 2.84]	Asparagine, ½-β-CH_2	2.0×10^{-187}
[2.40 .. 2.44]	Unassigned part-added Ni(II)- and Cu(II)-resistant multiplet, possibly superimposed on weak Pyruvate-CH_3 (*s*) and/or Succinate-CH_2 (*s*) resonances/Pyroglutamate-γ-CH_2	2.4×10^{-187}
[3.72 .. 3.76]	α-Glucose-C3H´	2.6×10^{-187}
[4.20 .. 4.24]	Pyroglutamate-α-CH_2s	3.2×10^{-187}
[5.28 .. 5.32]	α-Glucose-C1H	6.3×10^{-187}
[1.00 .. 1.04]	Leucine-CH_3/Isoleucine-γ-CH_3/Valine-CH_3	6.9×10^{-187}
[3.24 .. 3.28]	β-Glucose-C2H	8.1×10^{-187}
[0.96 .. 1.00]	Leucine-CH_3	8.5×10^{-187}
[2.08 .. 2.12]	Free O-Acetylated biomolecule-$OCOCH_3$	1.0×10^{-186}
[3.32 .. 3.36]	Methanol-CH_3	1.4×10^{-186}
[1.72 .. 1.76]	Lysine-δ-CH_2	1.5×10^{-186}
[3.04 .. 3.08]	Lysine-ε-CH_2	1.7×10^{-186}
[1.28 .. 1.32]	Possibly 3-Hydroxyisovalerate-CH_3	1.7×10^{-186}
[2.56 .. 2.60]	Methionine-γ-CH_2	3.2×10^{-186}
[2.48 .. 2.52]	Pyroglutamate-1/2-β-CH_2;/Hydroxyproline-1/2-β-CH_2	3.2×10^{-186}

Table S3. List of ranked ANOVA-derived *p* values showing the statistical significance of the top 30 discriminatory TSP-normalised metabolite buckets arising from the ^{1}H NMR titration of RPMI 1640 culture medium with increasing concentrations of Cu(II) ion (0-67 µmol/L). The TSP internal standard concentration was 497 µmol/L. The buckets arise from the 0.90-5.40 ppm chemical shift range only, and the FDR correction applied was Fisher's least significant difference test.

^{1}H NMR Bucket (ppm)	Assignment(s)	ANOVA FDR-Corrected *p* Value
[1.00 .. 1.04]	Leucine-CH_3/Isoleucine-γ-CH_3/Valine-CH_3	2.30×10^{-53}
[2.92 .. 2.96]	Asparagine, ½-β-CH_2	3.06×10^{-45}
[1.92 .. 1.96]	Acetate-CH_3	1.94×10^{-43}

[1.04 .. 1.08]	Valine-$C\underline{H}_3$	5.22×10^{-42}
[1.76 .. 1.80]	Lysine-δ-$C\underline{H}_2$	5.22×10^{-42}
[2.16 .. 2.20]	Methionine-$SC\underline{H}_3$	2.74×10^{-41}
[3.84 .. 3.88]	β-Glucose-C6$\underline{H}_a$	1.45×10^{-40}
[5.28 .. 5.32]	α-Glucose-C1$\underline{H}$	3.82×10^{-40}
[3.72 .. 3.76]	α-Glucose-C3$\underline{H}'$	1.42×10^{-38}
[0.96 .. 1.00]	Leucine-$C\underline{H}_3$	3.84×10^{-38}
[3.96 .. 4.00]	β-Glucose-C6$\underline{H}_a$	2.87×10^{-36}
[1.96 .. 2.00]	Arginine-γ-$C\underline{H}_2$	3.64×10^{-36}
[2.48 .. 2.52]	Pyroglutamate-1/2-β-$C\underline{H}_2$/Hydroxyproline-1/2-β-$C\underline{H}_2$	3.64×10^{-36}
[4.00 .. 4.04]	Cystine-$C\underline{H}$	2.13×10^{-35}
[3.88 .. 3.92]	β-Glucose-C6$\underline{H}_a$	4.62×10^{-35}
[1.72 .. 1.76]	Lysine-δ-$C\underline{H}_2$	1.40×10^{-34}
[1.68 .. 1.72]	Leucine-$C\underline{H}_2$	3.85×10^{-34}
[3.56 .. 3.60]	Glycine-$C\underline{H}_2$	7.36×10^{-34}
[3.28 .. 3.32]	β-Glucose-C2$\underline{H}$	2.98×10^{-33}
[3.80 .. 3.84]	α-Glucose-C6$\underline{H}'_b$	1.10×10^{-32}
[3.16 .. 3.20]	Choline-$N(C\underline{H}_3)_3^+$	5.20×10^{-32}
[3.08 .. 3.12]	Dimethylsulphone $SO(C\underline{H}_3)_2$	3.02×10^{-31}
[2.88 .. 2.92]	Asparagine, ½-β-CH_2	3.64×10^{-30}
[3.92 .. 3.96]	β-Glucose-C6$\underline{H}_a$	5.60×10^{-29}
[3.00 .. 3.04]	Lysine-ε-$C\underline{H}_2$	8.48×10^{-29}
[3.40 .. 3.44]	Methanol-$C\underline{H}_3$	9.00×10^{-29}
[2.44 .. 2.48]	Glutamine-γ-$C\underline{H}_2$	1.15×10^{-28}
[2.96 .. 3.00]	Asparagine, ½-β-CH_2	2.69×10^{-28}
[1.08 .. 1.12]	Unknown	1.61×10^{-27}
[3.76 .. 3.80]	α-Glucose-C6$\underline{H}'_b$	3.07×10^{-26}

Section S4: Further investigations of the ATR-FTIR Spectra of the Deposit Generated from the Interaction of Cu(II) with RPMI 1640 Culture Medium

Further ATR-FTIR investigations of the precise molecular nature of the deposit material generated on addition of Cu(II) to the culture medium featured in the current study, including the presence of any amino acids and/or other biomolecules, any Cu(II)-amino acid complexes, and/or its putative inorganic nanoparticulate Cu(II) base (copper(II)-oxides, -hydroxides, -carbonates (as in malachites) and/or -phosphates) core materials are further described herein (Figure S4). However, at this stage there is insufficient evidence to confirm the involvement of culture medium-donated amino acids, nor that of its possible or partial nanoparticulate nature. Therefore, this work will be continued, and reported elsewhere (further experiments are in progress).

The presence of amino acids in the solid material was also explored, and for this purpose we focussed on glutamine and arginine, which had the highest content in the RPMI 1640 culture medium (2.055 and 1.149 mol/L respectively). L-glutamine has FTIR absorption bands located at 2,800-3,000 (C-H stretch), 2,400-3,000 (N-H stretch), 2,041 (NH_3^+), 2,000-2,500 (combination bands of N-H bending/C-O rocking, plus triple bond-containing species at *ca.* 2,100), 1,650 (C=O stretch), 1,575 ($-NH_3^+$ symmetrical deformation), 1,000-1,400 (C-C, C-N, C-O stretches, carbon skeleton and side-chain), 400-500 (complex bending and rocking vibrations/various bonds) and 441 cm^{-1} (NH_3^+ torsion mode) [77]. However, L-arginine's absorption bands include those at 3,000-3,300 (broad, O-H and N-H stretching), 3,093 (C-H stretch), 2,857 (N-H stretch), 1,693 (C=O stretch), 1,610 (N-H deformation/bending), 1,577 cm^{-1} (N-H bending), and 1,130-1,155 cm^{-1} (C-N stretching) [78]. Hence, in view of a significant level of overlap with the dominant water bands, there appears to be only very limited evidence for the inclusion of culture medium amino acids in the added Cu(II)-induced deposit collected, although some relatively minor bands were visible in ATR-FTIR spectra of the untreated culture medium and that of its residual supernatant following removal of the deposit, and these were absent from the spectrum of the precipitate. These include quite sharp bands ascribable to glucose in the 990-1200 cm^{-1} region. Moreover, relatively weak bands located at 1,464 and 2,832 cm^{-1} in residual supernatant spectra only are assignable to FTIR-active functions in the HEPES buffer system [79].

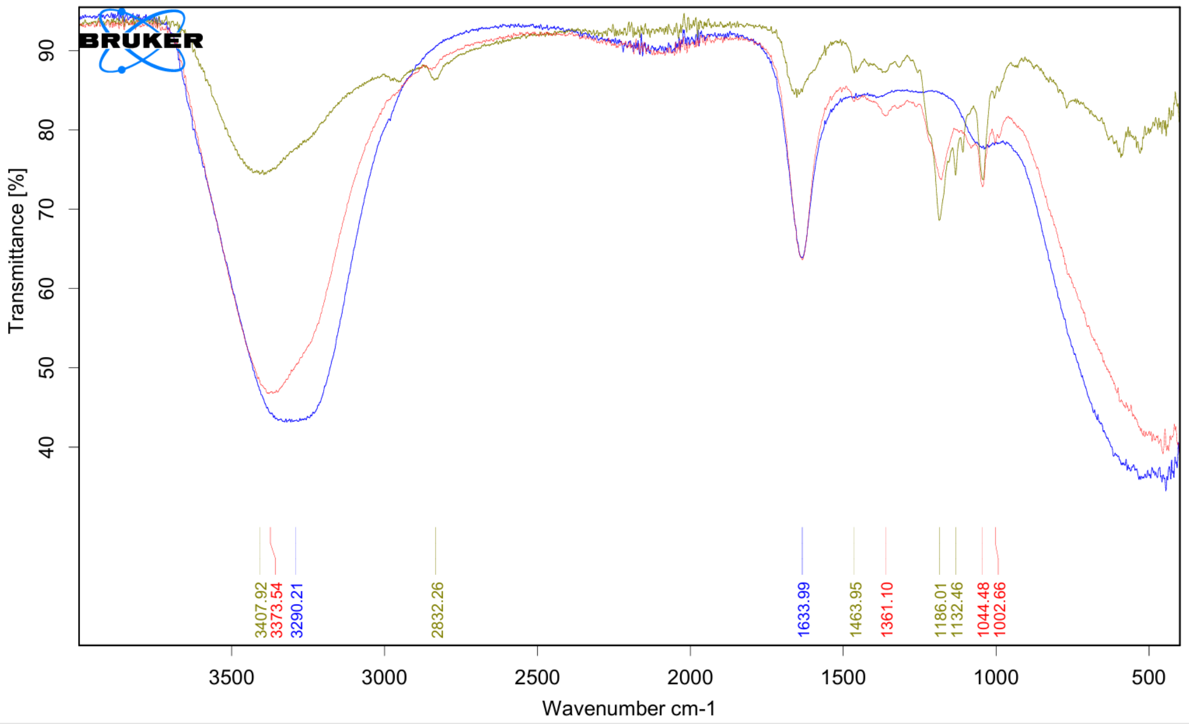

Figure S4. ATR-FTIR spectra of pale blue-coloured deposit arising from addition of Cu(II) to RPMI 1640 culture medium (blue), untreated culture medium (red) and residual culture medium supernatant following removal of the Cu(II)-induced precipitate (pale yellow-green). These spectra showed some differences between the Cu(II)-induced solid matrix produced and that of the untreated culture medium.

The known IR spectrum of the 1:1 Cu(II)-glutamine (Cu(II)-Gln) complex contains an absorption band in the 3150–3100 cm^{-1} region, and this corresponds to the stretching vibration of the uncoordinated -N-H group of the glutamine ligand [80]. Although this band was not visible in the Cu(II) deposit spectrum, nor in that of control (untreated) saliva, it was in that of the residual supernatant obtained following Cu(II) treatment. Unfortunately, it was not possible to check for co-ordinated carboxylate functions being present, as in the Cu(II) complexes of glutamine or arginine (the highest concentration amino acids present in the RPMI 1640 medium) in view of overlap from the intense water band at 1634 cm^{-1}. Indeed, this absorption band is typical for the asymmetric vibration of coordinated carboxylate groups, e.g., those of glutamine and arginine, along with other amino acids of lower culture medium concentration [80]. However, the absence of bands in the region of 1,660–1700 cm^{-1} of the deposit material spectrum suggests that there may be coordination of the -CO_2^- group of glutamine, arginine, or other amino acids, to the central Cu(II) ion. The band found around 1380-1400 cm^{-1} in the deposit spectrum (blue profile, Figure S4) could be assigned to the symmetric vibration of the coordinated carboxylate group. However, the diminished concentration of glutamine in the culture medium arising from its deterioration to pyroglutamate and ammonia (as visible in Figures 2 and 5) will clearly serve to retard the generation of any Cu(II)-Gln complexes. Similarly, there was little or no evidence for the uptake of further culture medium amino acids such as L-histidine or BCAAs by this pale blue-coloured deposit.

The FTIR spectral properties of the deposit also contain some broad absorption bands which do, however, provide some evidence, albeit limited, for the presence of inorganic, copper-containing agents in their composition, for example perhaps $Cu^{I}O$ (610-630 cm^{-1} [81,82]); $(Cu^{II})_3(PO_4)_2$ (3,421 (strong and broad, free O-H), 1,632 (O-H bend), (1,124 PO_4^{3-} stretch), 1,049 (P=O stretch), 752 (P-O-P system), and 618 cm^{-1} (Cu^{II}-O stretch) [83]; and/or $Cu^{II}CO_3$ (2,700-3,600, 1,485, 1,095 and 1,038 cm^{-1}) [84];).

Moreover, such Cu(II) compounds are known to display a very broad absorption from 550-700 cm^{-1}, as is indeed the case here, although the spectrum of 'neat' untreated samples also shows a superimposing 520 cm^{-1} water libration band (Figure S4).Characteristic Cu-O stretching vibrations for $Cu^{II}O$ and $Cu^{I}O$ are 536/586 cm^{-1}, and 621 cm^{-1} respectively [81].
